# When size makes a difference: allometry, life-history and morphological evolution of capuchins (*Cebus*) and squirrels (*Saimiri*) monkeys (Cebinae, Platyrrhini)

**DOI:** 10.1186/1471-2148-7-20

**Published:** 2007-02-14

**Authors:** Gabriel Marroig

**Affiliations:** 1 Departamento de Genética e Biologia Evolutiva, Instituto de Biociências, Universidade de São Paulo, CP 11.461, CEP 05422-970, São Paulo, Brasil

## Abstract

**Background:**

How are morphological evolution and developmental changes related? This rather old and intriguing question had a substantial boost after the 70s within the framework of heterochrony (changes in rates or timing of development) and nowadays has the potential to make another major leap forward through the combination of approaches: molecular biology, developmental experimentation, comparative systematic studies, geometric morphometrics and quantitative genetics. Here I take an integrated approach combining life-history comparative analyses, classical and geometric morphometrics applied to ontogenetic series to understand changes in size and shape which happen during the evolution of two New World Monkeys (NWM) sister genera.

**Results:**

*Cebus *and *Saimiri *share the same basic allometric patterns in skull traits, a result robust to sexual and ontogenetic variation. If adults of both genera are compared in the same scale (discounting size differences) most differences are small and not statistically significant. These results are consistent using both approaches, classical and geometric Morphometrics. *Cebus *is a genus characterized by a number of peramorphic traits (adult-like) while *Saimiri *is a genus with paedomorphic (child like) traits. Yet, the whole clade Cebinae is characterized by a unique combination of very high pre-natal growth rates and relatively slow post-natal growth rates when compared to the rest of the NWM. Morphologically Cebinae can be considered paedomorphic in relation to the other NWM. Geometric morphometrics allows the precise separation of absolute size, shape variation associated with size (allometry), and shape variation non-associated with size. Interestingly, and despite the fact that they were extracted as independent factors (principal components), evolutionary allometry (those differences in allometric shape associated with intergeneric differences) and ontogenetic allometry (differences in allometric shape associated with ontogenetic variation within genus) are correlated within these two genera. Furthermore, morphological differences produced along these two axes are quite similar. *Cebus *and *Saimiri *are aligned along the same evolutionary allometry and have parallel ontogenetic allometry trajectories.

**Conclusion:**

The evolution of these two Platyrrhini monkeys is basically due to a size differentiation (and consequently to shape changes associated with size). Many life-history changes are correlated or may be the causal agents in such evolution, such as delayed on-set of reproduction in *Cebus *and larger neonates in *Saimiri*.

## Background

Since Gould's publication of Ontogeny and Phylogeny [14] a wave of renewed interest in the role of development in generating evolutionary novelties spread through biology. Heterochrony, evolutionary modifications in the rates and/or the timing (onset and offset) of development [2], become widely recognized as an important agent of evolutionary change [26]. The study of heterochrony, while interesting per se, does not take us any closer to understanding the developmental, genetic, and physiological processes underlying evolutionary change [29]. Yet, the study of heterochrony, even when using size in place of time, may be quite helpful in understanding evolutionary diversification (see criticisms in [13]). Allometry, the differential and relative growth of organismal parts may be conceived as a size-based "heterochrony" [26]. Heterochrony addresses trait change relative to time and allometry examine trait change relative to others traits (usually size), the latter being a comparison of heterochronic results to one another [26].

Size and shape are important biological properties of organisms arising from their genetic basis in complex association and sometimes interaction with the external and internal environment. Usually, a large fraction of the variability in morphometric data is due to size variation among individuals. Scaling effects might result in shape changes associated with changing size due to allometric relationships among traits, unless all morphological components grow or scale at the same rates (isometry). A long tradition in morphometrics has been to regard size as a nuisance factor in comparisons of organisms with several methods being used to adjust size before comparisons (e.g. [3,39,32,20]). The rationale behind this approach is to regard size as a plastic feature of organisms and shape changes, unassociated with size (non-allometric), as adaptive [40]. Another motivation for developing methods allowing the separation of size and shape was the need to compare forms with very different sizes [41]. Yet, size is as much a property of organisms as is shape, with important functional and ecological implications. For example, a simple increase in skull size (and concomitant allometric shape changes) might result in larger animals being able to handle larger and harder food items and therefore explore new resources or niches. Here I present a study of size and shape variation in two New World primates, the squirrel (*Saimiri*) monkeys and capuchin (*Cebus*) monkeys. The approach used here combines traditional and geometric morphometrics, comparative analyses of life-history data and statistical analyses of size and shape differences to understand the evolution of these two sister genera.

The subfamily Cebinae, as used here, refers to the two modern genera, *Saimiri *and *Cebus*, which are united on the basis of dental morphology and proportions, overall cranial morphology and others skeletal features [9]. There is now a consensus that these two genera are indeed living sister clades based on recent phylogenetic studies [34,33,35]. Adult squirrel monkeys weigh less than 1.0 kg, on average (males 858 g and females 715 g) while capuchins usually weigh 3 times more (males 2,912 g and females 2,042 kg). Capuchins occur throughout the Neotropical region occupying virtually all types of forested habitats, from mangroves and disturbed forests to well-preserved Amazonian and Atlantic forests. Squirrel monkeys occur throughout the Amazon region to Central America, but not in the Cerrado and Atlantic forests, also occupying a great variety of forested habitats. Group sizes usually range from 6 to 30 individuals in *Cebus *while *Saimiri *had group size ranging from 10 to up to 75 individuals, and sometimes both genera mix together in foraging parties. Besides, both genera share some unique life-history patterns in New World Monkeys (NWM), with relatively heavy brains for their body weight [15].

Here I present a study of the morphological variation in *Cebus *and *Saimiri*, focusing on the allometric patterns, differentiation and evolution of size and shape in Cebinae. Ontogenetic and static allometric patterns and shape (free of size) variation are compared in order to describe similarities and differences in skull variation between genera. Finally, these results are compared to life-history traits and ecology of NWM to understand the Cebinae morphological evolution. All these approaches converge to a simple picture: Cebus and Saimiri evolved from a common ancestor basically diverging in size. This divergence follows a common ontogenetic trajectory which is basically revealed by the fact that evolutionary allometry (those differences in shape associated with size differences among lineages) and ontogentic allometry (shape changes associated with size differences during the ontogeny on each lineage) are highly correlated and morphologically describe the same changes in the skull. This size evolution might be caused by life-history changes like a delayed on-set of reproduction in Cebus.

## Results

### Static and ontogenetic allometry

Table 1 shows the multivariate allometric coefficients (ACs), corresponding standard deviations obtained from the bootstrap, and the lower and upper 95% confidence limits for each genus. Results for each sex analyzed separately are nearly equal to those presented here pooling both sexes within each genus and for simplicity are not presented. Those ACs with confidence limits not encompassing one (isometry) were considered either negatively (below 1) or positively (above 1) allometric. Eleven of the 17 neural traits (65%) and 9 of the 23 facial traits (39%) are negatively allometric in *Cebus *and the same figures for *Saimiri *are 13 in 17 (76% neural) and 11 in 23 (48% facial). Conversely, 2 of the 17 neural traits (12%) and 6 of the 23 facial traits (26%) are positively allometric in *Cebus *and the same figures for *Saimiri *are 3 in 17 (18% neural) and 8 in 23 (35% facial). Allometric vector repeatabilities are 0.99 for both genera and therefore sampling error is negligible in judging vector correlations. Allometric vector repeatabilities were also quite high in the sub-adult sample (t = 0.98 for *Saimiri *and t = 0.99 for *Cebus*) and therefore sampling error should have a negligible impact upon the vector similarities. The following vector correlations were obtained: *Saimiri *adult × *Cebus *adult = 0.968, *Cebus *adult × *Cebus *young = 0.978, *Saimiri *adult × *Cebus *young = 0.963, *Saimiri *young × *Saimiri *adult = 0.951, *Cebus *young × *Saimiri *young = 0.981, *Cebus *adult × *Saimiri *young = 0.980. Furthermore, the following averages and confidence interval were observed in the correlation of each vector against its 100 random permutation sample: *Saimiri *young = 0.773 (0.707–0.84), *Saimiri *adult = 0.82 (0.759–0.88), *Cebus *young = 0.808 (0.736–0.88), *Cebus *adult = 0.769 (0.697–0.842). Therefore all allometric vector correlations are higher that expected by the correlation of any two size vectors. Additionally, table 1 also show the PC1_total _extracted from the V/CV of the natural log-transformed data used in the MASS correction. This PC1 accounts for 90% of the total variation in the data and is quite similar (r = 0.954) to an isometric vector (all elements equal to 1/39^0.5^). Also, this PC1_total _is quite similar to the size vectors representing within genus variation (r = 0.936 with *Saimiri *and 0.912 with *Cebus*).

**Table 1:** Allometric coefficients

	Saimiri				Cebus					PC1_total_
Traits	AC	SE AC	L1	L2	AC	SE AC	L1	L2	Skull Region	

ISPM	**1.22**	0.07	1.09	1.35	***0.77***	0.07	0.63	0.90	Face	0.18
ISNSL	0.97	0.09	0.79	1.14	1.06	0.08	0.90	1.22	Face	0.16
ISPNS	***0.80***	0.08	0.64	0.95	***0.78***	0.07	0.65	0.91	Face	0.19
PMZS	0.85	0.09	0.67	1.03	1.06	0.08	0.91	1.21	Face	0.19
PMZI	***0.72***	0.10	0.53	0.90	0.95	0.10	0.76	1.14	Face	0.17
PMMT	***0.63***	0.05	0.54	0.73	***0.71***	0.06	0.60	0.82	Face	0.19
NSLNA	0.90	0.15	0.61	1.20	***0.64***	0.12	0.40	0.88	Face	0.20
NSLZS	0.89	0.08	0.73	1.04	***0.78***	0.06	0.66	0.89	Face	0.14
NSLZI	***0.77***	0.07	0.63	0.92	***0.86***	0.07	0.73	0.99	Face	0.14
NABR	***0.62***	0.06	0.50	0.74	***0.57***	0.07	0.43	0.70	Neurocranium	0.17
NAFM	***0.62***	0.05	0.53	0.72	***0.65***	0.05	0.55	0.74	Face	0.14
NAPNS	***0.62***	0.06	0.51	0.73	***0.84***	0.05	0.73	0.94	Face	0.16
BRPT	***0.50***	0.07	0.35	0.64	***0.42***	0.07	0.29	0.55	Neurocranium	0.16
BRAPET	***0.63***	0.05	0.53	0.74	***0.47***	0.04	0.38	0.56	Neurocranium	0.12
PTFM	***0.61***	0.18	0.25	0.96	0.90	0.33	0.26	1.55	Face	0.14
PTAPET	***0.65***	0.06	0.55	0.76	0.80	0.12	0.58	1.03	Neurocranium	0.14
PTBA	***0.89***	0.04	0.80	0.97	0.89	0.08	0.74	1.04	Neurocranium	0.15
PTEAM	1.12	0.07	0.98	1.26	1.12	0.11	0.90	1.34	Neurocranium	0.17
PTZYGO	**1.45**	0.09	1.27	1.64	**1.56**	0.14	1.29	1.84	Face	0.16
PTTSP	**1.39**	0.17	1.05	1.72	**1.82**	0.38	1.07	2.58	Neurocranium, face	0.13
FMZS	***0.42***	0.13	0.16	0.68	***0.44***	0.12	0.21	0.67	Face	0.11
FMMT	***0.86***	0.05	0.77	0.95	0.93	0.04	0.85	1.01	Face	0.17
ZSZI	***0.56***	0.10	0.37	0.76	0.85	0.12	0.61	1.08	Face	0.12
ZIMT	**1.43**	0.14	1.16	1.70	**1.47**	0.09	1.29	1.65	Face	0.25
ZIZYGO	**1.97**	0.15	1.67	2.27	**2.26**	0.11	2.04	2.48	Face	0.18
ZITSP	**1.53**	0.08	1.37	1.70	**1.64**	0.07	1.51	1.78	Face	0.15
MTPNS	***0.63***	0.06	0.51	0.74	0.88	0.07	0.74	1.02	Face	0.14
PNSAPET	**1.65**	0.14	1.37	1.93	**1.45**	0.09	1.26	1.63	Neurocranium	0.21
APETBA	**1.17**	0.08	1.01	1.34	1.08	0.05	0.98	1.18	Neurocranium	0.13
APETTS	***0.65***	0.08	0.50	0.81	***0.60***	0.07	0.45	0.74	Neurocranium	0.14
BAEAM	***0.84***	0.06	0.73	0.95	***0.66***	0.04	0.58	0.73	Neurocranium	0.16
EAMZYGO	**1.58**	0.16	1.28	1.89	0.90	0.10	0.70	1.10	Face	0.25
ZYGOTSP	**1.84**	0.10	1.65	2.03	**1.43**	0.07	1.29	1.57	Face	0.20
LDAS	***0.42***	0.09	0.23	0.60	***-0.16***	0.08	-0.31	0.00	Neurocranium	0.05
BRLD	***0.27***	0.08	0.13	0.42	***0.19***	0.18	-0.16	0.54	Neurocranium	-0.01
OPILD	***0.64***	0.17	0.30	0.98	***0.07***	0.11	-0.15	0.30	Neurocranium	0.06
PTAS	***0.82***	0.05	0.72	0.93	***0.87***	0.06	0.75	0.98	Neurocranium	0.17
JPAS	***0.76***	0.07	0.62	0.89	***0.68***	0.07	0.55	0.81	Neurocranium	0.13
BAOPI	***0.29***	0.11	0.07	0.50	***0.19***	0.07	0.05	0.34	Neurocranium	0.15

Multivariate allometry coefficients (AC), theirs standard errors (SE AC) and 95% confidence limits (L1 and L2) for both genera based on the first principal component extracted from each genus within-group V/CV matrix. PC1 vectors were normalized and each coefficient divided by (1/39)^1/2 ^to obtain the AC. Standard deviation estimates obtained from bootstrap analysis. Allometric coefficients with L1 higher that one (isometry) were considered to be positively allometric with general size (shown in bold and underlined) and conversely, AC with L2 lower that one were considered to be negatively allometric (bold and italic) with size. ACs with confidence limits encompassing 1.0 were considered to be isometric with size (normal font). The last column show the first principal component extracted from the whole sample (*Cebus*+*Saimiri*) and used in the MASS transformation.

### Differentiation with and without size

A MANOVA was performed on the 39 measurements using sex, genus, and sex by genus interaction as independent variables in order to determine whether sexual dimorphism needs to be accounted for in the analyses. Five hundred sixty-four individuals were analyzed and significant multivariate (Wilk's Λ = 0.016; df = 39, 522; *P *< 1.0 × 10^-5^) and univariate (all *P *< 1.0 × 10^-4^) differences between the genera were found. There was also significant multivariate differences in sex (Wilk's Λ = 0.462; df = 39, 522; *P *< 1.0 × 10^-5^). Thirty-five variables presented univariate differences in sex significant at P < 1.0 × 10^-3^, two were significant between 1% and 5% (BA-OPI and OPI-LD) and two were found non-significant (LD-AS and BR-LD). Moreover, there was also significant multivariate sex by genus interaction (Wilk's Λ = 0.741; df = 39, 522; *P *< 1.0 × 10^-5^) and 33 significant sex by genus interaction (31 with P < 0.001 and 2 with P < 0.05) in the univariate tests. There is strong evidence for differentiation between the two genera, the two sexes and for the interaction of sex with genus. Additionally, an ANOVA performed on size (first principal component extracted from the V/CV matrix of the LN transformed data accounting for 90% of the total variance) show significant differences between the two genera (MS_genus _= 530.27, F = 14228.63, P > 10^-5^), between the two sexes (MS_sex _= 10.70, F = 287.21, P > 10^-5^) and also significant interaction between effects (MS_genus*sex _= 0.39, F = 10.48, P = 0.001), all effects with 1 degree of freedom (error term with d.f = 560 and MS_error _= 0.037). Therefore all analyses below were done independently for both sexes, except where specifically noted, allowing also to properly control for interespecific variation within each genus.

**Figure 1:**
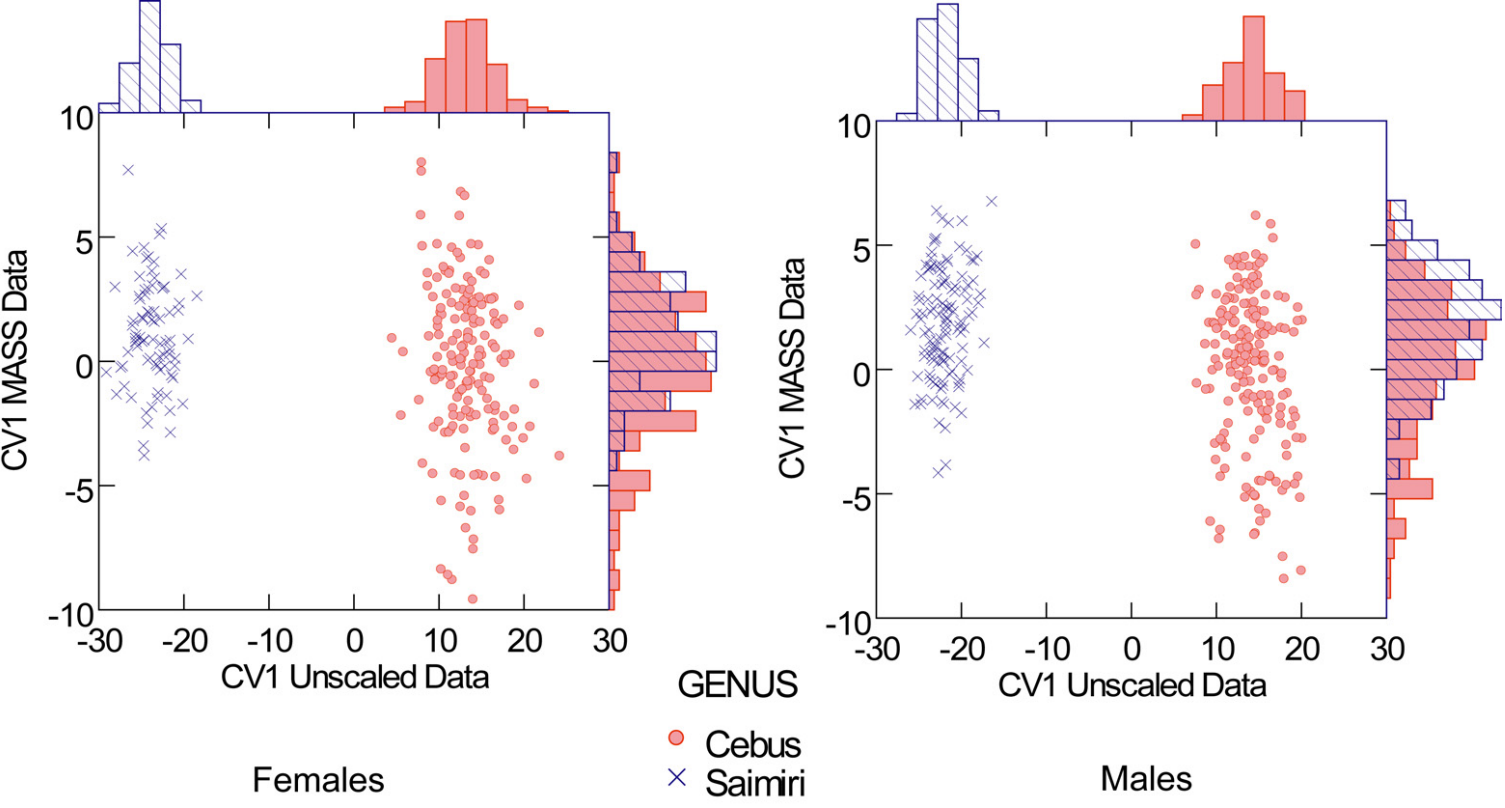
**Canonical variates**. Canonical variate 1 (CV1) obtained separately for males and females with frequency distribution of CV1 scores on the margin. On the x-axis is shown the CV1 obtained from the unscaled data and on the y-axis the CV1 from the MASS corrected data.

The MANOVA performed on the original unscaled variables using 309 complete male skulls with genera and species nested within genera as factors was highly significant (Wilk's Λ = 0.021; df = 39, 252; *P *< 0.0001) with the single canonical variate (Table 2) separating completely the two groups (Figure 1). Correlations between CV scores and skull measurements are also presented in Table 2. Based on the correlations between variables and function, the CV is a size factor because all the significant correlations are positive, except for BR-LD. CV has large contributions from both neurocranial and facial traits. The MANOVA performed on the 255 complete female skulls with genera and species nested within genera as factors was also highly significant (Wilk's Λ = 0.032; df = 39, 198; *P *< 0.0001) with the single CV (Table 2) also separating the two groups completely (Figure 1). Correlations between CV scores and skull measurements are also presented in Table 2. The two CV's (males and females) are very similar with a vector correlation between them of 0.90. The MANOVA results with species nested within genus indicate that only one trait (BR-LD) does not show significant differences between the two genera (using the conservative Bonferroni correction of the significance level P = 0.05/39) in the univariate F-tests for both, males and females (Table 3).

**Table 2:** Canonical variate functions and correlations of traits to function

	Canonical Variate		Correlation between traits and function			Canonical Variate		Correlation between traits and function	
	Males	Females	Males	Females		Males	Females	Males	Females

Traits	CV1 original	CV1 original	CV1 original	CV1 original	Traits	CV1 MASS	CV1 MASS	CV1 MASS	CV1 MASS

ISPM	-0.154	0.023	**0.963**	**0.968**	MASS-ISPM	-0.225	-0.467	0.161	0.168
ISNSL	0.136	-0.085	**0.936**	**0.930**	MASS-ISNSL	0.059	-0.639	0.078	**0.291**
ISPNS	0.383	0.725	**0.978**	**0.970**	MASS-ISPNS	-0.469	-0.792	**-0.209**	-0.024
PMZS	-0.047	-0.067	**0.954**	**0.951**	MASS-PMZS	-0.967	0.549	**0.282**	**0.488**
PMZI	-0.159	-0.136	**0.938**	**0.951**	MASS-PMZI	0.585	-1.334	**0.301**	**0.471**
PMMT	0.577	0.384	**0.986**	**0.979**	MASS-PMMT	-0.252	-0.119	-0.104	**0.323**
NSLNA	-0.070	0.100	**0.901**	**0.899**	MASS-NSLNA	0.368	-0.781	0.138	0.109
NSLZS	0.461	0.668	**0.949**	**0.947**	MASS-NSLZS	0.397	-1.054	**0.227**	**0.440**
NSLZI	-0.854	-1.495	**0.950**	**0.958**	MASS-NSLZI	-0.581	1.321	**0.251**	**0.418**
NABR	-0.056	-0.136	**0.972**	**0.959**	MASS-NABR	0.534	-0.623	**-0.211**	**-0.464**
NAFM	0.314	0.483	**0.959**	**0.952**	MASS-NAFM	-0.442	-0.333	**-0.345**	-0.161
NAPNS	-0.036	-0.195	**0.973**	**0.967**	MASS-NAPNS	-0.581	-0.222	-0.140	0.135
BRPT	0.129	-0.365	**0.973**	**0.956**	MASS-BRPT	-1.026	0.010	**-0.213**	**-0.404**
BRAPET	0.197	0.639	**0.971**	**0.955**	MASS-BRAPET	0.095	-0.102	-0.152	**-0.363**
PTFM	0.664	0.553	**0.765**	**0.649**	MASS-PTFM	-1.705	-1.057	0.099	0.114
PTAPET	-1.479	-2.920	**0.946**	**0.940**	MASS-PTAPET	-2.141	-0.645	**-0.468**	**-0.378**
PTBA	2.578	3.363	**0.973**	**0.978**	MASS-PTBA	1.585	-0.118	**-0.274**	**-0.284**
PTEAM	-0.050	0.088	**0.953**	**0.959**	MASS-PTEAM	0.300	-0.205	**-0.249**	**-0.281**
PTZYGO	-0.438	0.921	**0.897**	**0.904**	MASS-PTZYGO	0.025	-0.899	-0.145	**-0.295**
PTTSP	-0.314	-1.039	**0.714**	**0.648**	MASS-PTTSP	-1.337	-0.494	**-0.387**	**-0.389**
FMZS	0.451	0.534	**0.840**	**0.823**	MASS-FMZS	-0.877	-0.546	**-0.257**	**-0.271**
FMMT	-0.050	-0.098	**0.974**	**0.981**	MASS-FMMT	0.295	-0.147	0.104	0.180
ZSZI	0.069	0.602	**0.875**	**0.870**	MASS-ZSZI	0.152	-1.053	**0.192**	0.190
ZIMT	0.176	0.350	**0.946**	**0.964**	MASS-ZIMT	-0.615	-0.586	**0.353**	**0.452**
ZIZYGO	-0.792	-0.960	**0.816**	**0.807**	MASS-ZIZYGO	0.250	-0.740	**0.237**	0.026
ZITSP	-0.063	-0.247	**0.886**	**0.883**	MASS-ZITSP	-0.202	-0.689	0.167	-0.107
MTPNS	0.131	-0.016	**0.928**	**0.945**	MASS-MTPNS	-0.472	-0.418	-0.087	-0.006
PNSAPET	-0.110	0.424	**0.919**	**0.933**	MASS-PNSAPET	-0.329	-0.586	**0.338**	**0.218**
APETBA	-0.854	-1.075	**0.930**	**0.944**	MASS-APETBA	-1.021	-0.512	**0.273**	0.106
APETTS	0.128	0.139	**0.932**	**0.932**	MASS-APETTS	-0.215	-0.387	-0.010	0.042
BAEAM	-0.247	-0.077	**0.983**	**0.975**	MASS-BAEAM	-0.107	-0.157	-0.100	-0.087
EAMZYGO	-0.247	-0.286	**0.955**	**0.934**	MASS-EAMZYGO	-0.130	-1.242	**-0.263**	-0.196
ZYGOTSP	-0.073	-0.360	**0.931**	**0.955**	MASS-ZYGOTSP	-0.698	-0.584	**0.303**	0.057
LDAS	-0.082	0.096	**0.648**	**0.721**	MASS-LDAS	-0.076	-0.539	**-0.363**	**-0.617**
BRLD	-0.125	-0.494	-0.105	-0.208	MASS-BRLD	0.159	0.221	0.130	**0.360**
OPILD	-0.088	-0.398	**0.606**	**0.599**	MASS-OPILD	-0.293	-0.402	**-0.378**	**-0.617**
PTAS	0.747	0.718	**0.980**	**0.982**	MASS-PTAS	-0.961	-0.702	**-0.405**	**-0.250**
JPAS	-0.036	-0.053	**0.946**	**0.944**	MASS-JPAS	-0.011	-0.129	0.053	0.208
BAOPI	0.162	0.102	**0.934**	**0.923**	MASS-BAOPI	-0.398	-0.460	**-0.345**	0.023

The canonical variate obtained for both males and females using either unscaled or MASS data are show. Also the correlation of each trait to each CV is also show, with significant (P < 0.05) correlations in bold.

**Table 3:** Differentiation analyses results from unscaled data

Males						Females					
Source	SS	df	MS	F	P	Source	SS	df	MS	F	P

ISPM	624.03	1	624.03	1881.23	**< 0.00001**	ISPM	270.34	1	270.34	1589.58	**< 0.00001**
Error	96.20	290	0.33			Error	40.14	236	0.17		
ISNSL	2208.91	1	2208.91	1094.03	**< 0.00001**	ISNSL	753.22	1	753.22	561.51	**< 0.00001**
Error	585.52	290	2.02			Error	316.57	236	1.34		
ISPNS	8866.98	1	8866.98	3443.39	**< 0.00001**	ISPNS	3466.56	1	3466.56	1878.82	**< 0.00001**
Error	746.77	290	2.58			Error	435.44	236	1.85		
PMZS	2771.68	1	2771.68	1988.35	**< 0.00001**	PMZS	904.62	1	904.62	799.20	**< 0.00001**
Error	404.25	290	1.39			Error	267.13	236	1.13		
PMZI	4907.99	1	4907.99	1624.74	**< 0.00001**	PMZI	1768.39	1	1768.39	921.21	**< 0.00001**
Error	876.03	290	3.02			Error	453.03	236	1.92		
PMMT	6091.46	1	6091.46	5727.18	**< 0.00001**	PMMT	2282.79	1	2282.79	2606.01	**< 0.00001**
Error	308.45	290	1.06			Error	206.73	236	0.88		
NSLNA	1614.81	1	1614.81	653.76	**< 0.00001**	NSLNA	678.68	1	678.68	279.02	**< 0.00001**
Error	716.31	290	2.47			Error	574.05	236	2.43		
NSLZS	1600.16	1	1600.16	1552.98	**< 0.00001**	NSLZS	566.57	1	566.57	650.30	**< 0.00001**
Error	298.81	290	1.03			Error	205.61	236	0.87		
NSLZI	5429.01	1	5429.01	1596.46	**< 0.00001**	NSLZI	2000.24	1	2000.24	891.50	**< 0.00001**
Error	986.19	290	3.40			Error	529.51	236	2.24		
NABR	19412.50	1	19412.50	2202.66	**< 0.00001**	NABR	7782.90	1	7782.90	875.87	**< 0.00001**
Error	2555.83	290	8.81			Error	2097.06	236	8.89		
NAFM	2352.00	1	2352.00	2073.69	**< 0.00001**	NAFM	856.69	1	856.69	1166.02	**< 0.00001**
Error	328.92	290	1.13			Error	173.39	236	0.73		
NAPNS	6079.51	1	6079.51	2707.97	**< 0.00001**	NAPNS	2159.91	1	2159.91	1270.51	**< 0.00001**
Error	651.06	290	2.25			Error	401.21	236	1.70		
BRPT	11044.23	1	11044.23	2274.23	**< 0.00001**	BRPT	4500.39	1	4500.39	727.55	**< 0.00001**
Error	1408.31	290	4.86			Error	1459.82	236	6.19		
BRAPET	6227.39	1	6227.39	2353.72	**< 0.00001**	BRAPET	2522.35	1	2522.35	916.62	**< 0.00001**
Error	767.27	290	2.65			Error	649.42	236	2.75		
PTFM	848.11	1	848.11	293.76	**< 0.00001**	PTFM	259.66	1	259.66	101.66	**< 0.00001**
Error	837.26	290	2.89			Error	602.80	236	2.55		
PTAPET	3520.35	1	3520.35	896.99	**< 0.00001**	PTAPET	1273.51	1	1273.51	363.74	**< 0.00001**
Error	1138.14	290	3.92			Error	826.28	236	3.50		
PTBA	9388.69	1	9388.69	1681.36	**< 0.00001**	PTBA	3645.97	1	3645.97	751.50	**< 0.00001**
Error	1619.35	290	5.58			Error	1144.97	236	4.85		
PTEAM	6012.35	1	6012.35	1120.82	**< 0.00001**	PTEAM	2257.54	1	2257.54	491.86	**< 0.00001**
Error	1555.63	290	5.36			Error	1083.19	236	4.59		
PTZYGO	3409.62	1	3409.62	524.15	**< 0.00001**	PTZYGO	1225.50	1	1225.50	238.55	**< 0.00001**
Error	1886.46	290	6.51			Error	1212.40	236	5.14		
PTTSP	570.13	1	570.13	122.77	**< 0.00001**	PTTSP	166.37	1	166.37	35.31	**< 0.00001**
Error	1346.77	290	4.64			Error	1111.99	236	4.71		
FMZS	725.73	1	725.73	511.53	**< 0.00001**	FMZS	319.79	1	319.79	236.29	**< 0.00001**
Error	411.43	290	1.42			Error	319.40	236	1.35		
FMMT	6504.36	1	6504.36	2604.76	**< 0.00001**	FMMT	2440.20	1	2440.20	1724.24	**< 0.00001**
Error	724.16	290	2.50			Error	334.00	236	1.42		
ZSZI	1124.79	1	1124.79	578.97	**< 0.00001**	ZSZI	436.54	1	436.54	277.55	**< 0.00001**
Error	563.39	290	1.94			Error	371.19	236	1.57		
ZIMT	3466.72	1	3466.72	1490.40	**< 0.00001**	ZIMT	1179.17	1	1179.17	1336.39	**< 0.00001**
Error	674.55	290	2.33			Error	208.23	236	0.88		
ZIZYGO	1513.75	1	1513.75	275.46	**< 0.00001**	ZIZYGO	470.49	1	470.49	136.15	**< 0.00001**
Error	1593.66	290	5.50			Error	815.52	236	3.46		
ZITSP	2221.52	1	2221.52	512.02	**< 0.00001**	ZITSP	826.68	1	826.68	363.06	**< 0.00001**
Error	1258.23	290	4.34			Error	537.36	236	2.28		
MTPNS	599.94	1	599.94	1097.34	**< 0.00001**	MTPNS	207.63	1	207.63	722.52	**< 0.00001**
Error	158.55	290	0.55			Error	67.82	236	0.29		
PNSAPET	1931.14	1	1931.14	763.05	**< 0.00001**	PNSAPET	701.46	1	701.46	431.80	**< 0.00001**
Error	733.94	290	2.53			Error	383.38	236	1.62		
APETBA	1194.94	1	1194.94	816.34	**< 0.00001**	APETBA	472.17	1	472.17	551.26	**< 0.00001**
Error	424.50	290	1.46			Error	202.14	236	0.86		
APETTS	843.27	1	843.27	1062.46	**< 0.00001**	APETTS	320.89	1	320.89	540.82	**< 0.00001**
Error	230.17	290	0.79			Error	140.03	236	0.59		
BAEAM	3155.44	1	3155.44	3416.08	**< 0.00001**	BAEAM	1217.39	1	1217.39	1465.18	**< 0.00001**
Error	267.87	290	0.92			Error	196.09	236	0.83		
EAMZYGO	4471.57	1	4471.57	1638.73	**< 0.00001**	EAMZYGO	1645.18	1	1645.18	696.06	**< 0.00001**
Error	791.32	290	2.73			Error	557.80	236	2.36		
ZYGOTSP	1901.08	1	1901.08	851.07	**< 0.00001**	ZYGOTSP	739.99	1	739.99	722.40	**< 0.00001**
Error	647.79	290	2.23			Error	241.74	236	1.02		
LDAS	491.17	1	491.17	229.61	**< 0.00001**	LDAS	308.24	1	308.24	153.01	**< 0.00001**
Error	620.34	290	2.14			Error	475.43	236	2.01		
BRLD	2.03	1	2.03	0.16	0.69321	BRLD	27.08	1	27.08	2.54	0.11255
Error	3781.79	290	13.04			Error	2519.18	236	10.67		
OPILD	650.73	1	650.73	149.32	**< 0.00001**	OPILD	454.45	1	454.45	97.59	**< 0.00001**
Error	1263.76	290	4.36			Error	1098.99	236	4.66		
PTAS	13504.40	1	13504.40	2474.10	**< 0.00001**	PTAS	5011.51	1	5011.51	1031.10	**< 0.00001**
Error	1582.91	290	5.46			Error	1147.04	236	4.86		
JPAS	1382.67	1	1382.67	1147.25	**< 0.00001**	JPAS	519.56	1	519.56	596.60	**< 0.00001**
Error	349.51	290	1.21			Error	205.52	236	0.87		
BAOPI	907.95	1	907.95	1342.30	**< 0.00001**	BAOPI	316.07	1	316.07	474.52	**< 0.00001**
Error	196.16	290	0.68			Error	157.20	236	0.67		

Results from the GLM analyses for each sex using unscaled data with "genus" as a factor and "species" nested within genus as independent factors. For each trait is presented the Sum of Squares (SS), degrees of freedom (df), Mean Square (MS), F value and the associated probability (P). Significant differences in bold.

Results from the MANOVA done upon the MASS corrected data are quite different from the analyses upon the original unscaled data. While the CV (Table 2) is also highly significant for males (Wilk's Λ = 0.413; df = 39, 252; P < 0.0001) the two genera are now widely overlapping (Figure 1). The same pattern holds for females, with the CV (Table 2) being also significant (Wilk's Λ = 0.642; df = 39, 198; P < 0.0001), the scores of the two groups widely overlapping (Figure 1). Moreover, correlations of the variables with the CV, for both males and females, are now very small with around half of them being significant (Table 2). The MANOVA results with species nested within genus shows only two traits (IS-PNS and PM-ZS) with significant difference for the females (again using the Bonferroni correction) and six traits with significant differences in the males (IS-PNS, NA-FM, NA-PNS, PT-FM, ZI-ZYGO, PNS-APET, Table 4).

**Table 4:** Differentiation analyses results from MASS data

Males						Females					
Source	SS	df	MS	F	P	Source	SS	df	MS	F	P

MASS-ISPM	0.06	1	0.06	0.38	0.53703	MASS-ISPM	1.76	1	1.76	12.94	0.00039
Error	45.32	290	0.16			Error	32.07	236	0.14		
MASS-ISNSL	7.30	1	7.30	6.06	0.01442	MASS-ISNSL	0.10	1	0.10	0.09	0.76821
Error	349.42	290	1.20			Error	272.50	236	1.15		
MASS-ISPNS	47.66	1	47.66	33.05	**<0.00001**	MASS-ISPNS	31.42	1	31.42	19.92	**0.00001**
Error	418.21	290	1.44			Error	372.15	236	1.58		
MASS-PMZS	0.37	1	0.37	0.46	0.49812	MASS-PMZS	16.80	1	16.80	18.23	**0.00003**
Error	232.91	290	0.80			Error	217.43	236	0.92		
MASS-PMZI	2.87	1	2.87	1.63	0.20299	MASS-PMZI	0.13	1	0.13	0.09	0.76245
Error	511.86	290	1.77			Error	325.44	236	1.38		
MASS-PMMT	6.26	1	6.26	5.20	0.02333	MASS-PMMT	1.45	1	1.45	1.73	0.19019
Error	349.16	290	1.20			Error	197.64	236	0.84		
MASS-NSLNA	22.70	1	22.70	10.06	0.00168	MASS-NSLNA	9.87	1	9.87	4.43	0.03637
Error	654.58	290	2.26			Error	525.89	236	2.23		
MASS-NSLZS	5.11	1	5.11	7.82	0.0055	MASS-NSLZS	7.14	1	7.14	9.10	0.00283
Error	189.32	290	0.65			Error	185.17	236	0.78		
MASS-NSLZI	1.31	1	1.31	0.91	0.34022	MASS-NSLZI	0.00	1	0.00	0.00	0.94965
Error	415.99	290	1.43			Error	278.84	236	1.18		
MASS-NABR	9.64	1	9.64	1.34	0.24798	MASS-NABR	3.59	1	3.59	0.44	0.50744
Error	2086.87	290	7.20			Error	1921.51	236	8.14		
MASS-NAFM	13.38	1	13.38	18.94	**0.00002**	MASS-NAFM	1.39	1	1.39	2.16	0.14339
Error	204.90	290	0.71			Error	152.70	236	0.65		
MASS-NAPNS	22.52	1	22.52	19.03	**0.00002**	MASS-NAPNS	1.43	1	1.43	1.39	0.239
Error	343.23	290	1.18			Error	241.62	236	1.02		
MASS-BRPT	0.57	1	0.57	0.11	0.73811	MASS-BRPT	0.05	1	0.05	0.01	0.92523
Error	1472.11	290	5.08			Error	1303.78	236	5.52		
MASS-BRAPET	4.26	1	4.26	2.44	0.11953	MASS-BRAPET	2.68	1	2.68	1.25	0.265
Error	506.40	290	1.75			Error	506.94	236	2.15		
MASS-PTFM	50.68	1	50.68	22.99	**<0.00001**	MASS-PTFM	5.97	1	5.97	2.45	0.11876
Error	639.43	290	2.20			Error	575.23	236	2.44		
MASS-PTAPET	0.04	1	0.04	0.02	0.90209	MASS-PTAPET	2.64	1	2.64	1.10	0.29604
Error	731.79	290	2.52			Error	567.86	236	2.41		
MASS-PTBA	8.29	1	8.29	3.53	0.06136	MASS-PTBA	1.62	1	1.62	0.61	0.43607
Error	681.78	290	2.35			Error	627.62	236	2.66		
MASS-PTEAM	22.87	1	22.87	9.07	0.00282	MASS-PTEAM	3.63	1	3.63	1.26	0.2624
Error	731.02	290	2.52			Error	679.63	236	2.88		
MASS-PTZYGO	33.73	1	33.73	10.53	0.00132	MASS-PTZYGO	6.35	1	6.35	1.81	0.18004
Error	929.47	290	3.21			Error	828.24	236	3.51		
MASS-PTTSP	13.38	1	13.38	3.84	0.05094	MASS-PTTSP	10.85	1	10.85	2.70	0.10192
Error	1009.69	290	3.48			Error	949.35	236	4.02		
MASS-FMZS	7.68	1	7.68	6.71	0.01005	MASS-FMZS	6.68	1	6.68	5.29	0.02235
Error	331.62	290	1.14			Error	298.05	236	1.26		
MASS-FMMT	4.20	1	4.20	7.69	0.0059	MASS-FMMT	1.64	1	1.64	2.72	0.10044
Error	158.15	290	0.55			Error	142.66	236	0.60		
MASS-ZSZI	3.67	1	3.67	2.76	0.09755	MASS-ZSZI	1.79	1	1.79	1.41	0.23645
Error	384.99	290	1.33			Error	299.18	236	1.27		
MASS-ZIMT	0.08	1	0.08	0.10	0.75682	MASS-ZIMT	0.17	1	0.17	0.28	0.59791
Error	247.42	290	0.85			Error	144.36	236	0.61		
MASS-ZIZYGO	54.67	1	54.67	16.68	**0.00006**	MASS-ZIZYGO	24.54	1	24.54	8.44	0.00402
Error	950.39	290	3.28			Error	686.25	236	2.91		
MASS-ZITSP	1.28	1	1.28	0.58	0.44756	MASS-ZITSP	0.45	1	0.45	0.28	0.5991
Error	640.34	290	2.21			Error	381.88	236	1.62		
MASS-MTPNS	3.03	1	3.03	10.24	0.00153	MASS-MTPNS	0.03	1	0.03	0.14	0.70912
Error	85.76	290	0.30			Error	47.70	236	0.20		
MASS-PNSAPET	49.00	1	49.00	40.10	**<0.00001**	MASS-PNSAPET	14.42	1	14.42	11.31	0.0009
Error	354.33	290	1.22			Error	300.94	236	1.28		
MASS-APETBA	0.30	1	0.30	0.36	0.54778	MASS-APETBA	0.42	1	0.42	0.62	0.43181
Error	243.43	290	0.84			Error	159.95	236	0.68		
MASS-APETTS	3.02	1	3.02	4.59	0.03291	MASS-APETTS	0.49	1	0.49	0.95	0.3315
Error	190.37	290	0.66			Error	121.68	236	0.52		
MASS-BAEAM	0.73	1	0.73	1.61	0.20485	MASS-BAEAM	0.02	1	0.02	0.05	0.82879
Error	131.42	290	0.45			Error	109.71	236	0.46		
MASS-EAMZYGO	13.68	1	13.68	8.35	0.00416	MASS-EAMZYGO	22.44	1	22.44	9.83	0.00194
Error	475.33	290	1.64			Error	539.08	236	2.28		
MASS-ZYGOTSP	1.71	1	1.71	1.77	0.18456	MASS-ZYGOTSP	3.04	1	3.04	4.20	0.04151
Error	279.83	290	0.96			Error	170.80	236	0.72		
MASS-LDAS	9.86	1	9.86	4.67	0.03149	MASS-LDAS	27.25	1	27.25	13.63	0.00028
Error	611.85	290	2.11			Error	472.02	236	2.00		
MASS-BRLD	49.78	1	49.78	3.68	0.05594	MASS-BRLD	0.08	1	0.08	0.01	0.93036
Error	3919.26	290	13.51			Error	2551.68	236	10.81		
MASS-OPILD	15.31	1	15.31	3.61	0.0583	MASS-OPILD	56.06	1	56.06	11.81	0.0007
Error	1228.41	290	4.24			Error	1120.04	236	4.75		
MASS-PTAS	0.61	1	0.61	0.22	0.64043	MASS-PTAS	0.80	1	0.80	0.33	0.56576
Error	815.43	290	2.81			Error	574.13	236	2.43		
MASS-JPAS	0.01	1	0.01	0.01	0.91656	MASS-JPAS	0.27	1	0.27	0.41	0.52287
Error	258.37	290	0.89			Error	154.58	236	0.65		
MASS-BAOPI	5.46	1	5.46	6.39	0.01198	MASS-BAOPI	0.01	1	0.01	0.02	0.89451
Error	247.63	290	0.85			Error	182.77	236	0.77		

Results from the GLM analyses for each sex using MASS data with "genus" as a factor and "species" nested within genus as independent factors. For each trait is presented the Sum of Squares (SS), degrees of freedom (df), Mean Square (MS), F value and the associated probability (P). Significant differences in bold.

Interestingly, the MANOVA performed upon the MASS corrected data to test for genus, sex and sex by genus effects shows only a small interaction of the factors, with only 3 traits (IS-PM, PM-ZS and LD-AS) deemed significant. Also, few traits are significant between genera (ZI-ZYGO, BR-PT, PM-MT). Conversely, 17 of the 39 traits show significance differences between the sexes using the conservative Bonferroni threshold.

### Heterochrony and life-history

**Figure 2:**
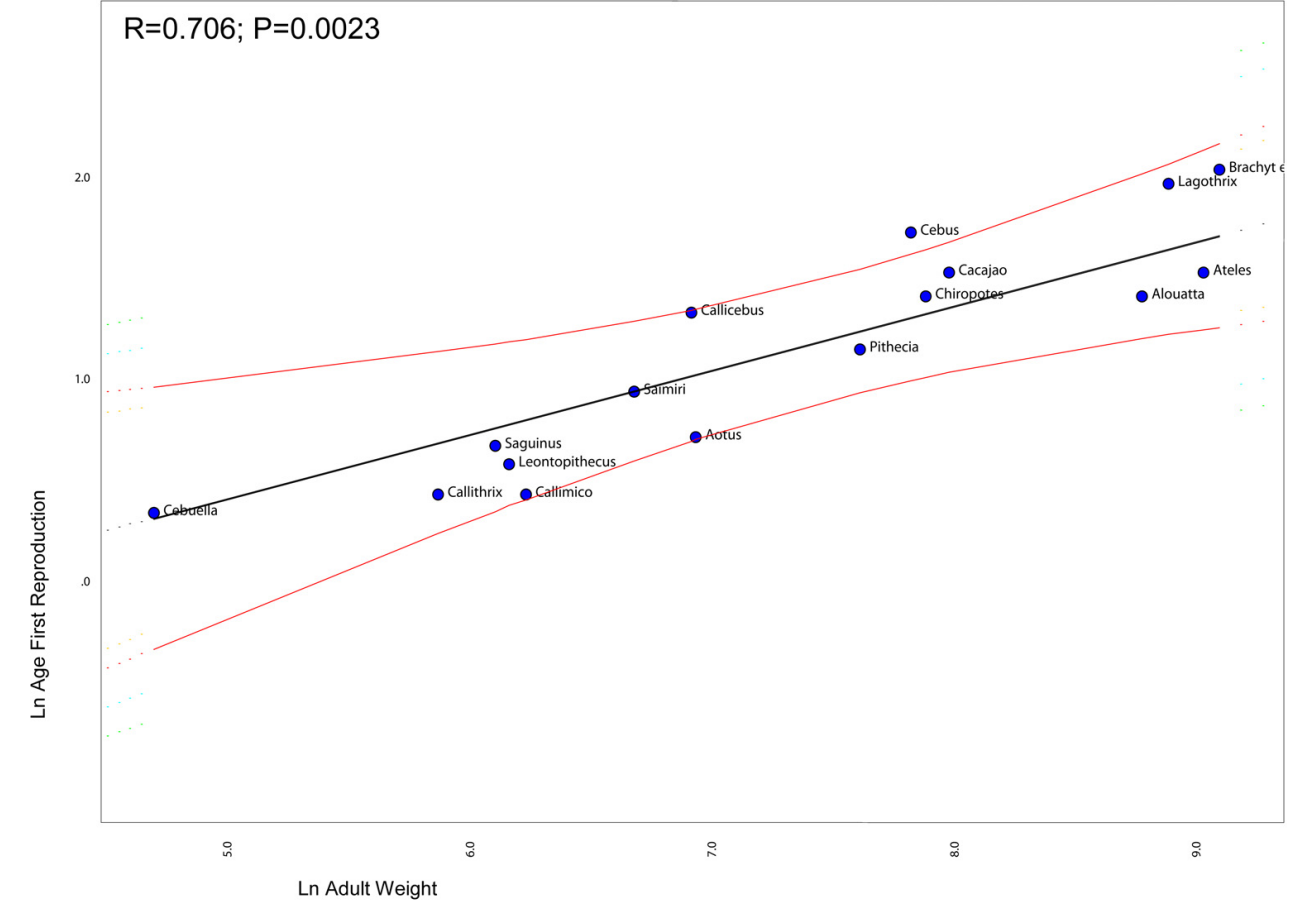
**Comparative phylogenetic regression of life-history traits**. Plot of the age of first reproduction against adult weight in New World Monkeys controlling for shared history (phylogeny). The regression line and 95% confidence limits were obtained from the method described in Garland and Ives (2000) and implemented in package PDAP in MESQUITE.

**Figure 3:**
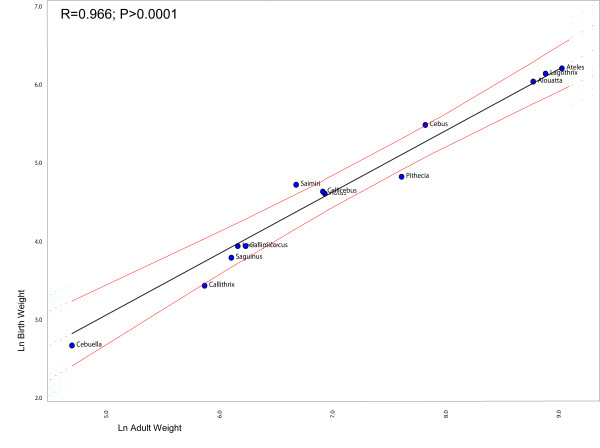
**Comparative phylogenetic regression of life-history traits**. Plot of the birth weight against adult weight in New World Monkeys controlling for shared history (phylogeny). The regression line and 95% confidence limits were obtained from the method described in Garland and Ives (2000) and implemented in package PDAP in MESQUITE.

**Figure 4:**
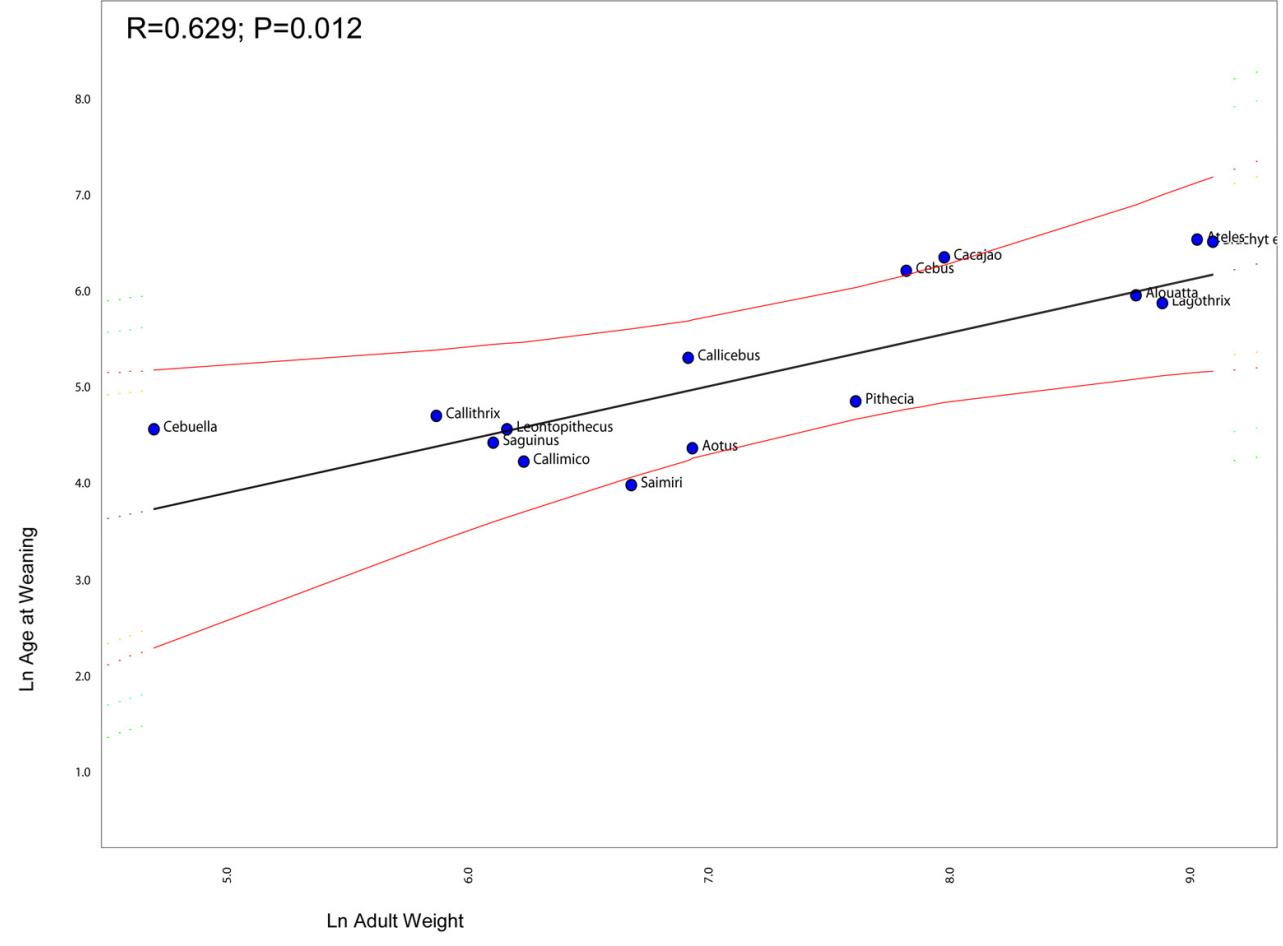
**Comparative phylogenetic regression of life-history traits**. Plot of the age at weaning against adult weight in New World Monkeys controlling for shared history (phylogeny). The regression line and 95% confidence limits were obtained from the method described in Garland and Ives (2000) and implemented in package PDAP in MESQUITE.

**Figure 5:**
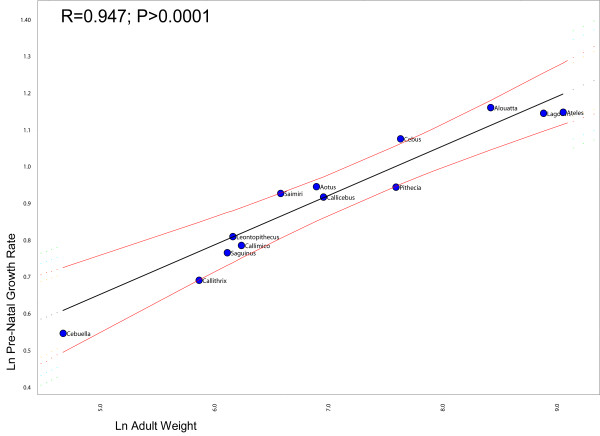
**Comparative phylogenetic regression of life-history traits**. Plot of the pre-natal growth rate against adult weight in New World Monkeys controlling for shared history (phylogeny). The regression line and 95% confidence limits were obtained from the method described in Garland and Ives (2000) and implemented in package PDAP in MESQUITE.

Figure 2 shows the regression between the ages of first reproduction against adult weights, after correcting for non-independence between points due to shared history (phylogeny). Notice that *Cebus *is the only genus deviating significantly from the regression line. This indicates that capuchins have a delayed on-set of reproduction in relation to the other genera given that its age of first reproduction is larger than expected for a NWM of its size. Likewise, Figure 3 shows the regression between the birth weights against body weight (the result is the same if skull size is used instead of body weight). Notice that squirrel monkeys deviate significantly from the regression line. This indicates that *Saimiri *babies are born heavier than expected for a NWM with its body size. Figure 4 shows the regression of the age at weaning against adult body weight. Squirrels monkeys seem to lie slightly below the 95% confidence interval of the regression line indicating that they are weaned earlier than expected for a NWM of its size. Conversely, capuchins seem to deviate from the regression line in the upper direction, suggesting that they are weaned later that expected for a NWM of its size. Figure 5 show the regression of the fetal growth rate (birth weight/gestation length) against adult body weight. *Saimiri *and *Cebus *lie slightly above the regression line. These patterns seem to be robust to within genus between species variation in life-history data. Unfortunately, complete information on life-history traits is not available for all species within each genus as well as solid phylogenetic hypotheses for all species within each of the two genera.

### Growth trajectories

I tested the assumption of size as a proxy for age by regressing size (PC1) against developmental age codes ([30], ages 1 to 6) separately for males and females. *Cebus *data was used because the juveniles and sub-adult sampling is much more extensive in this genus and have sex information available. Both regressions were highly significant (P < 0.00001) and the multiple R was 0.80 for females and 0.82 for males. Similar analyses in *Saimiri *(but ignoring sex dimorphism due to lack of sex in most of the juvenile and sub-adult sample) show also a similar multiple R (0.81). Furthermore, for *Cebus apella *at least there is available information for absolute age (in months) for each age class based on dental eruption (see Table 2 in [30]). Therefore is possible to calculate the correspondence between absolute size, time and age classes. Age classes and the natural log of age (in months) present a correlation of 0.97 for males of *Cebus apella*. Absolute time and size are also highly correlated (0.82) again indicating that size is a reasonable proxy to time. Given that absolute age is not available for the genus *Saimiri*, I use dental age classes here in the paper as a "developmental marker" and absolute size as an estimate of time.

**Figure 6:**
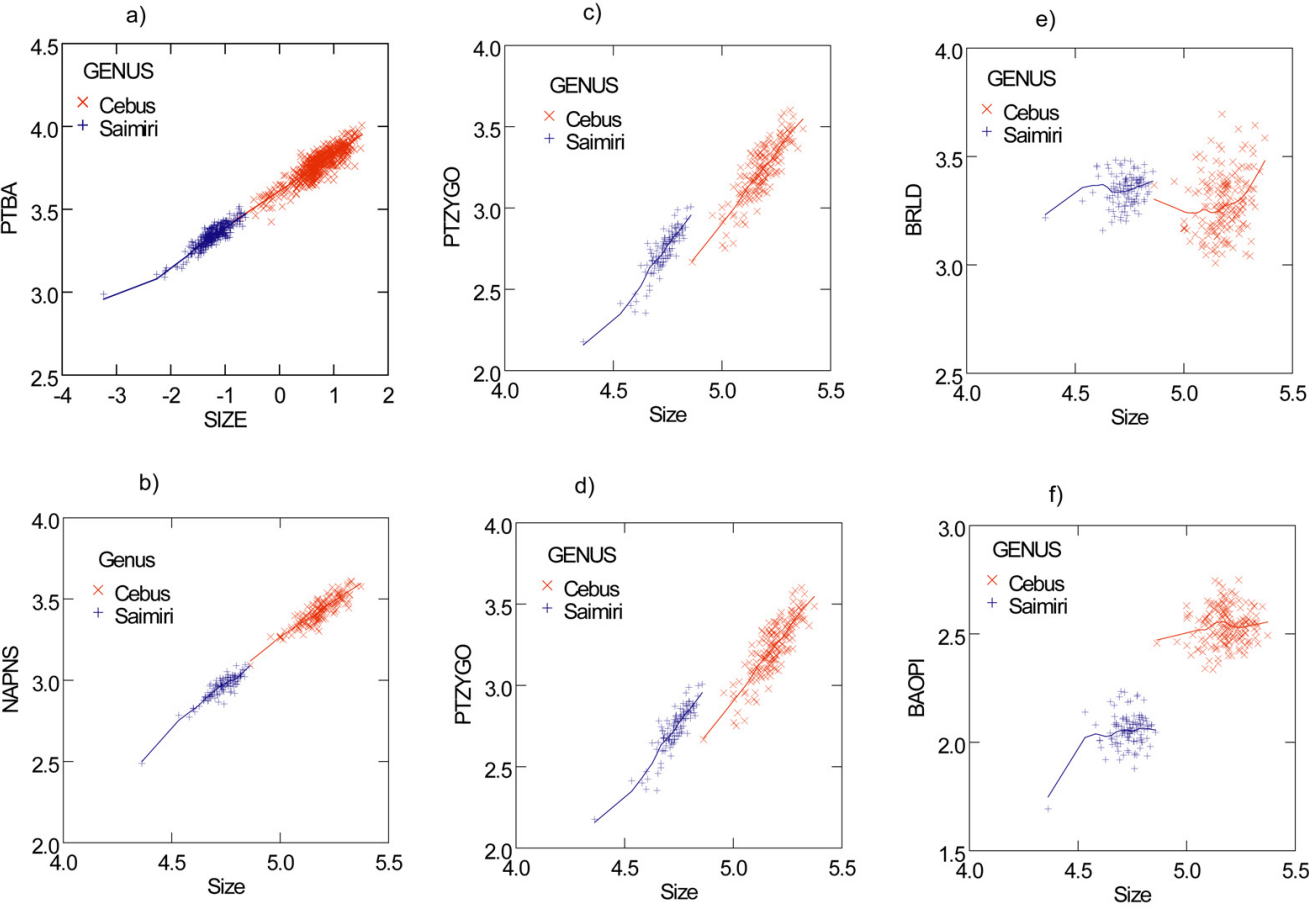
**Growth trajectories**. Bivariate plots of 6 skull measurements against centroid size (both in ln). A and B correspond to simple extension pattern, C and D to the step pattern, E to the low correlation with size pattern and F to the only trait showing an apparent mix of extension/step patterns (see also Table 5). The fit correspond to the LOWESS function with *Saimiri *in blue and *Cebus *in red.

**Figure 7:**
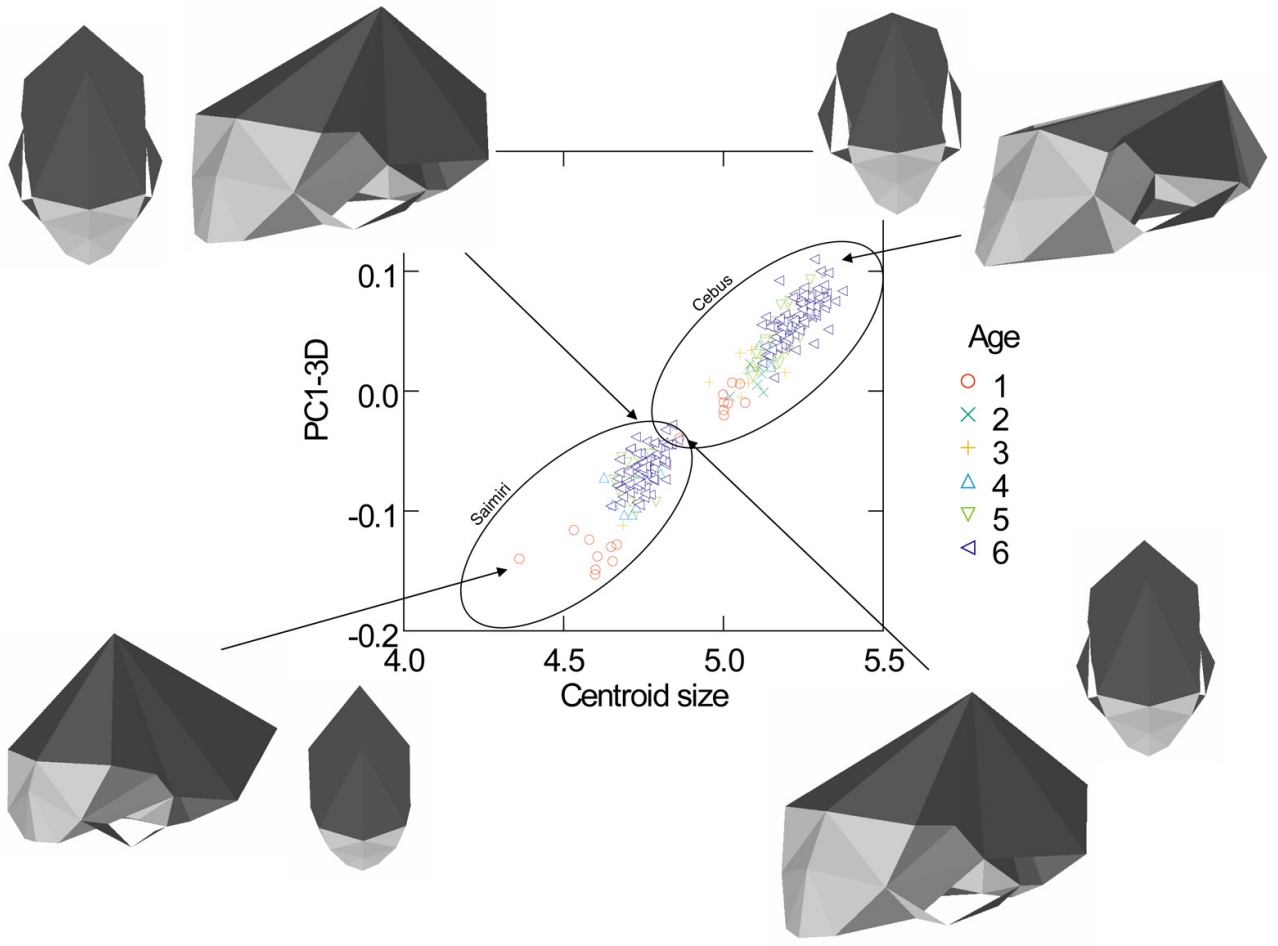
**Geometric Morphometrics – size and evolutionary shape allometry**. Plot of the PC1-3D against centroid size (ln). Specimens with different ages are show in different colours and symbols and skull 3D reconstructions are show in oblique and dorsal views.

Comparison of the growth trajectories are summarized in Figure 6 and Table 5. Most traits present a linear trajectory in the ln-scale, but some exceptions occur. Two general patterns are evident: 1) simple extension/truncation of the growth trajectory (Fig. 7a, 7b) 2) a step along the size (time) axis causing a shift in the otherwise parallel trajectories with *Saimiri *above (Fig. 7c, d). Most traits conform to one of these two patterns with a few exceptions. A group of such exceptions correspond to those traits which present very low correlation with size (Table 5) including traits LD-AS, BR-LD, OPI-LD, BA-OPI (Fig. 7e,f). Twenty-six traits (67% of all traits) can be assigned to pattern 1 (simple extension) and nine traits (23%) to pattern 2 (step along the size axis) (see Table 5).

**Table 5:** Growth trajectories

Trait	Pattern 1	Pattern 2	Functional/developmental group
ISPM	hypermorphosis/progenesis		Oral
ISNSL		pre-/postdisplacement (Saimiri above)	Nasal
ISPNS	hypermorphosis/progenesis		Oral, nasal
PMZS	hypermorphosis/progenesis		Oral
PMZI	hypermorphosis/progenesis		Oral
PMMT	hypermorphosis/progenesis		Oral
NSLNA	hypermorphosis/progenesis		Nasal
NSLZS	hypermorphosis/progenesis		Nasal
NSLZI	hypermorphosis/progenesis		Oral, nasal
NABR	hypermorphosis/progenesis		Cranial vault
NAFM	hypermorphosis/progenesis		Orbit
NAPNS	hypermorphosis/progenesis		Nasal
BRPT	hypermorphosis/progenesis		Cranial vault
BRAPET	hypermorphosis/progenesis		Cranial vault
PTFM		pre-/postdisplacement (Saimiri above)	Orbit
PTAPET	hypermorphosis/progenesis		Cranial vault
PTBA	hypermorphosis/progenesis		Cranial vault
PTEAM	hypermorphosis/progenesis		Cranial vault
PTZYGO		pre-/postdisplacement (Saimiri above)	Zygomatic
PTTSP		pre-/postdisplacement (Saimiri above)	Cranial vault, zygomatic
FMZS	hypermorphosis/progenesis		Orbit
FMMT	hypermorphosis/progenesis		Zygomatic
ZSZI		pre-/postdisplacement (Saimiri above)	Oral
ZIMT	hypermorphosis/progenesis		Oral
ZIZYGO		pre-/postdisplacement (Saimiri above)	Zygomatic
ZITSP		pre-/postdisplacement (Saimiri above)	Zygomatic
MTPNS	hypermorphosis/progenesis		Oral
PNSAPET	hypermorphosis/progenesis		Cranial base
APETBA		pre-/postdisplacement (Saimiri above)	Cranial base
APETTS	hypermorphosis/progenesis		Cranial base
BAEAM	hypermorphosis/progenesis		Cranial base
EAMZYGO	hypermorphosis/progenesis		Zygomatic
ZYGOTSP		pre-/postdisplacement (Saimiri above)	Zygomatic
LDAS	low correlation with size	low correlation with size	Cranial vault
BRLD	low correlation with size	low correlation with size	Cranial vault
OPILD	low correlation with size	low correlation with size	Cranial vault
PTAS	hypermorphosis/progenesis		Cranial vault
JPAS	hypermorphosis/progenesis		Cranial base
BAOPI	low correlation with size	low correlation with size	Cranial base

Interpretation of the bivariate plots (each trait against centroid size) relative to heterochronic processes.

### Geometric morphometrics

**Figure 8:**
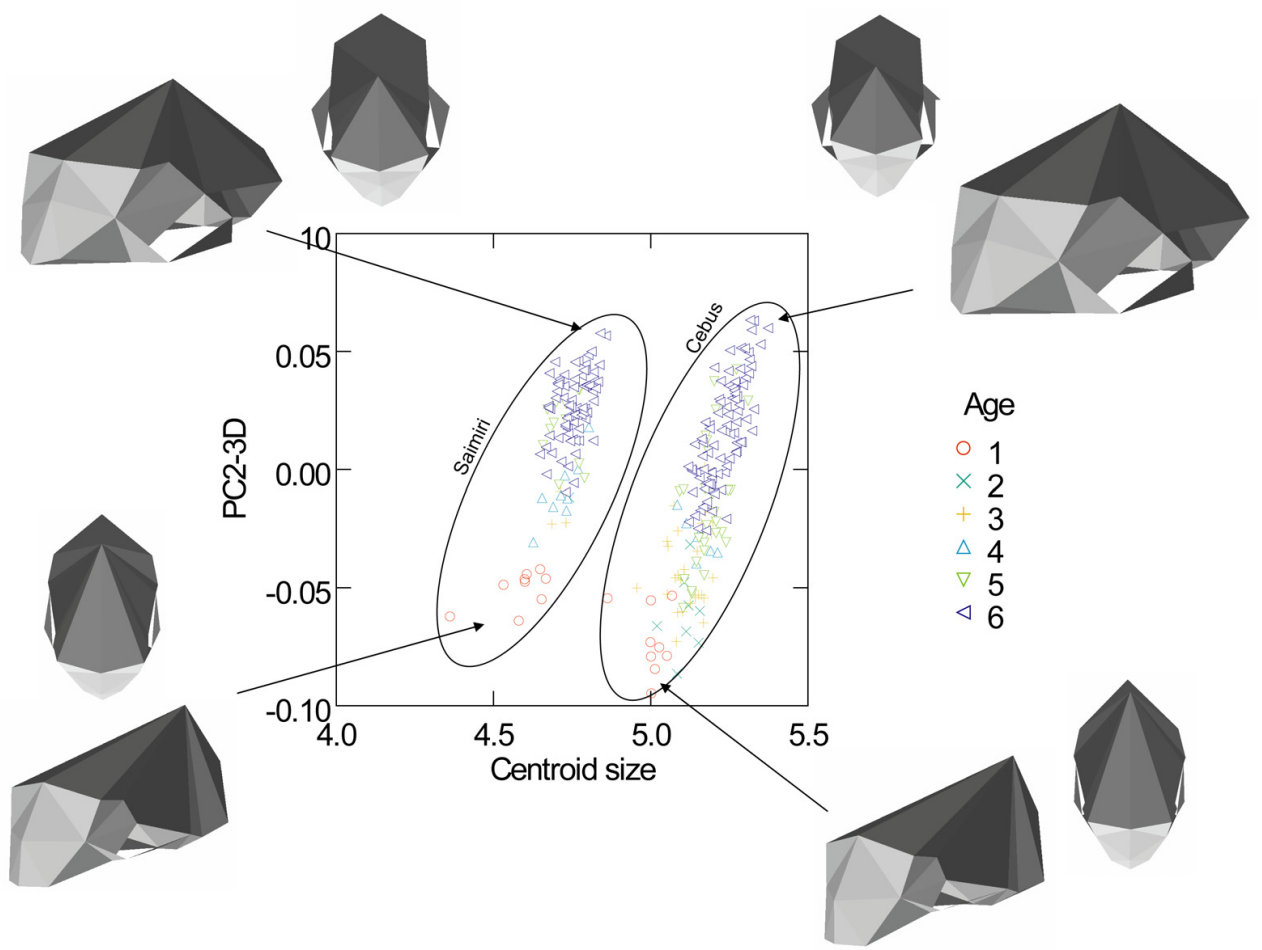
**Geometric Morphometrics – size and ontogenetic shape allometry**. Plot of the PC2-3D against centroid size (ln). Specimens with different ages are show in different colours and symbols and skull 3D reconstructions are show in oblique and dorsal views.

Centroid size is highly correlated with PC1 score derived from the linear distances (R = 0.999, P < 0.0001). Also, centroid size and PC1 score are linearly related when both are in ln scale. Therefore I use the natural log of the centroid size as a measure of absolute skull size. Figure 7 show the plot of the first PC-3D against centroid size [see Additional files 1, 2, 3, 4, 5, 6, 7, 8, 9]. This PC1-3D basically represents allometric variation in shape associated with size, accounting for 51.5% of all variation in shape. Starting from the smaller values (*Saimiri *young's), morphologically PC1-3D represents a lowering of the cranial vault with a large dislocation of landmark BR to a more posterior position as well as a relatively smaller posterior cranial base with a large dislocation of landmark LD to a more anterior position. Therefore, a major change described by this PC1-3D is that involving the neurocranium, with changes in the height of the vault associated with the base/back of the skull, involving landmarks LD, BR and to a lesser extent AS. These landmarks are dislocated from a more posterior (LD) and lateral (AS) position in *Saimiri *babies to a more anterior (LD) and medial position (AS) in *Cebus *adults. Those changes in landmarks BR and LD dominates the PC1-3D which can be easily observed from a vector of changes in landmarks coordinates comparing the upper and higher limits of variation described by PC1-3D (Table 6). Another change in shape associated with PC1-3D is in the face, being more prognathic (landmarks IS and PM) in the upper end (*Cebus*), with a longer and slender palate (landmark MT) and the zygomatic arch (more robust and lateral – landmarks ZI and ZYGO). Also associated with this PC1-3D is the dislocation of landmarks PT and TSP to a more medial position resulting in a more slender skull in *Cebus *(Figure 7). Figure 8 present the plot of the second PC-3D against size. This PC2-3D is basically an ontogenetic vector accounting for 15.6% of all variation in shape. The PC2-3D represents (again starting from the smaller values – *Cebus *and *Saimiri *young's) a relative decrease in the neurocranium region with landmark BR once more involved but this time being dislocated to a forward and lower position. Also, an enhanced prognathism resulting from landmarks IS and PM being dislocated forward and upward. Another change involves landmarks MT and ZI being dislocated forward and to a lower position resulting in a palate region comparatively smaller, face more prognathic and with a more robust pre-zygomatic region. Also, the cranial base is to some extent relatively smaller with landmark TSP being dislocated to a more posterior position and closer to landmarks APET, BA, TS and JP. Contributing to PC2-3D is also, and again, a dislocation of landmarks LD to a more anterior position and AS to a more medial position, exactly the same change described in PC1-3D. So, to some extent changes in shape described by PC1-3D and PC2-3D are similar (Table 6).

Only the PC1-3D presents significant differences between *Cebus *and *Saimiri *(t = 53.97, df = 231.6, P < 10^-5^) and this difference holds for all age classes analyzed separately. All other PC's variables, that cumulatively account for 97% of all shape variation (from PC2 to PC 40) do not present any significant differences between the two genera.

**Table 6:** Landmarks change vectors

	Saimiri					Cebus						
Landmarks	Ages 1–2	Ages 2–3	Ages 3–4	Ages 4–5	Ages 5–6	Ages 1–2	Ages 2–3	Ages 3–4	Ages 4–5	Ages 5–6	PC1-3D	PC2-3D

IS	**0.234**	**0.229**	**0.217**	**0.220**	**0.204**	**0.215**	**0.196**	**0.205**	**0.204**	**0.188**	**0.083**	0.044
PM(E)	**0.253**	**0.248**	**0.244**	**0.236**	**0.235**	**0.233**	**0.238**	**0.232**	**0.226**	**0.231**	0.055	**0.084**
NSL	0.029	0.031	0.082	0.021	0.103	0.033	0.136	0.090	0.065	0.128	0.017	0.071
NA	0.026	0.027	0.048	0.031	0.057	0.034	0.070	0.052	0.041	0.067	0.006	0.013
BR	**0.357**	**0.365**	**0.397**	**0.367**	**0.415**	**0.381**	**0.408**	**0.415**	**0.412**	**0.429**	**0.691**	**0.931**
PT(E)	0.192	0.193	0.205	0.199	0.209	0.193	0.208	0.207	0.203	0.211	**0.102**	0.056
FM(E)	0.031	0.031	0.016	0.044	0.009	0.042	0.022	0.014	0.034	0.015	0.013	0.006
ZS(E)	0.080	0.074	0.074	0.075	0.067	0.073	0.076	0.062	0.066	0.059	0.011	0.012
ZI(E)	**0.263**	**0.259**	**0.237**	**0.262**	**0.235**	**0.251**	**0.249**	**0.228**	**0.221**	**0.233**	**0.098**	0.075
MT(E)	**0.236**	**0.235**	**0.241**	**0.230**	**0.243**	**0.232**	**0.255**	**0.236**	**0.233**	**0.243**	0.035	**0.148**
PNS	0.102	0.101	0.088	0.099	0.081	0.096	0.070	0.083	0.088	0.070	0.014	0.009
APET(E)	0.129	0.127	0.095	0.133	0.080	0.126	0.052	0.090	0.107	0.061	0.040	0.007
BA	0.190	0.186	0.204	0.172	0.204	0.175	0.217	0.198	0.181	0.204	0.008	**0.101**
OPI	0.086	0.084	0.083	0.082	0.080	0.081	0.077	0.080	0.079	0.076	0.006	0.006
EAM(E)	0.169	0.170	0.171	0.177	0.173	0.175	0.173	0.171	0.167	0.173	0.079	0.044
PEAM(E)	0.199	0.198	0.217	0.188	0.221	0.193	0.229	0.216	0.204	0.224	0.035	0.063
ZYGO(E)	**0.284**	**0.280**	**0.316**	**0.258**	**0.322**	**0.265**	**0.346**	**0.310**	**0.283**	**0.328**	0.039	**0.163**
TSP(E)	0.143	0.145	0.184	0.142	0.204	0.148	0.228	0.194	0.172	0.226	0.067	**0.156**
TS (E)	0.152	0.151	0.161	0.146	0.162	0.149	0.163	0.161	0.155	0.163	0.036	0.030
JP(E)	0.101	0.099	0.096	0.099	0.096	0.100	0.089	0.098	0.101	0.093	0.017	0.013
LD	**0.464**	**0.471**	**0.409**	**0.498**	**0.389**	**0.492**	**0.345**	**0.418**	**0.469**	**0.374**	**0.681**	**0.120**
AS (E)	**0.309**	**0.308**	**0.296**	**0.307**	**0.288**	**0.306**	**0.266**	**0.293**	**0.300**	**0.274**	**0.097**	**0.076**

For each consecutive age (age 1 and 2) and for each of the two genera the vector of absolute change in landmark position is show. All vectors were normalized to one in order to be directly comparable. Also, the magnitude of change in landmark position between the two extremes is each of the allometric vectors (PC1-3D and PC2-3D) is show.

Both PC1-3D and PC2-3D are highly correlated with size variation within each genus (Table 7). Also, PC1-3D and PC2-3D are also highly correlated between them within each of the two genera. PC1-3D is also highly correlated with size among genera (R = 0.98, P < 10^-5^), while PC2-3D scores are uncorrelated (R close to zero) with both size and PC1-3D among genera (as expected because PC1 and PC2 are by definition extracted as orthogonal vectors). All other PC's variables are uncorrelated with size (from PC3 to PC 40).

**Table 7:** Correlation between absolute size, evolutionary and ontogenetic allometry

	SIZE	PC1-3D	PC2-3D
SIZE	1	P < 10^-5^	0.320
PC1-3D	0.972	1	0.872
PC2-3D	-0.060	0.009	1
Cebus	SIZE	PC1-3D	PC2-3D
SIZE	1	P < 10^-5^	P < 10^-5^
PC1-3D	0.864	1	P < 10^-5^
PC2-3D	0.809	0.768	1
Saimiri	SIZE	PC1-3D	PC2-3D
SIZE	1	P < 10^-5^	P < 10^-5^
PC1-3D	0.723	1	P < 10^-5^
PC2-3D	0.699	0.868	1

The Pearson correlation and associated probability between absolute size, PC1-3D and PC2-3D are show for a conjoint analysis (both genera) and for each of the two genera.

## Discussion

Sexual dimorphism in *Cebus *and *Saimiri *is well marked, either in the original traits or the MASS corrected data. Indeed, 33 of the unscaled traits show significant sexual dimorphism in *Cebus *and 30 in *Saimiri*, using the conservative Bonferroni threshold. After removing scale differences, MASS corrected data show 17 traits with significant sexual dimorphism in *Cebus *and 11 in *Saimiri *(again using the 0.05/39 threshold). Males in both genera are larger than females, but skull size dimorphism is more evident in *Cebus *(on average females are 66% of the males size) while in *Saimiri *females are on average 82% of the males. Besides, both sexes share a high similarity in their allometric vector correlation (0.948 in *Cebus *and 0.945 in *Saimiri*). Altogether these results suggests that sexual dimorphism in Cebinae is not simply a function of size related differences. In other words, if females were to grow to the same size as males in either *Saimiri *or *Cebus*, sexual dimorphism in shape would still be evident. Therefore nearly all analyses were performed separately for each sex.

Differences between the two genera are massive (Figure 1) considering the original data, with Mahalanobis D^2 ^distances pointing out the complete separation of the two groups in both sexes (D^2^_males _= 1299 and D^2^_females _= 1374). Conversely, there is a wide overlap between both genera considering the MASS corrected data (Figure 1) with very low D^2 ^distances (D^2^_males _= 3.84 and D^2^_females _= 1.74). Moreover, 38 of the 39 original traits show significant differences (P > 10^-5^) between the two genera in both sexes. Conversely, only two traits show significant differences between the two genera after correcting for scaling differences (MASS data) in females. Males present a slightly larger differentiation with 6 traits showing significant differences between the genera in the MASS corrected data. Taken together these results suggest that most of the differences between *Cebus *and *Saimiri *are related to size. Indeed, the only trait in the original scale not showing significant differences between the two genera (BR-LD) is the only one not influenced by size (Table 1 PC1_total_). This is an interesting result given that these two landmarks BR and LD are by far the most influential in the shape changes described by PC1-3D and PC2-3D. In fact, given that PC1-3D is the axis of major differentiation between *Saimiri *and *Cebus*, and that BR is dislocated to a more posterior and lower position while LD is dislocated forward that explain why the linear distance between the two landmarks is basically the same in both genera, despite the huge size difference between them. This can also be observed in the additional on-line material [see Additional files 1, 2, 3, 4, 5, 6, 7, 8, 9], particularly on the lateral view. After removing scale differences from the data (MASS correction) the large differences between the two genera nearly disappear, with only a small differentiation being observed. These results from the Euclidean distances analyses are totally consistent with the results obtained from the geometric morphometrics approach. Moreover, these results also show that males are somewhat more differentiated than females, after removing scale differences. In short, for the most part, squirrel monkeys are scaled down versions of capuchins, or vice versa.

Allometric vectors are much more similar than expected by chance in all comparisons as indicated by the comparisons of observed vectors correlations against its random permutations. This can be quantified by the angles formed between those allometric vectors, with observed angles ranging from 11.18° to 18.01°, well below the minimum expected angle of 28.36 ° from the random permutations. Given strong similarity in the allometric vectors of *Cebus *and *Saimiri*, and that most of the differences between them are size-related, it is seems clear that during the evolutionary diversification of these two sister genera size plays a major role. Conservation of allometric patterns in *Cebus *and *Saimiri *suggest that they simply follow the same growth patterns but evolved to attain different adult final sizes (Figure 6a, b). This conclusion is reinforced by the results of the geometric Morphometrics approach where the absolute magnitude of changes in landmarks position along PC1-3D and PC2-3D between consecutive age classes within *Saimiri *and *Cebus *are compared. All vectors of change are similar either within or between genus and present vectors correlation above 0.97, which again is well above the expected range from the random permutation tests (0.60–0.87).

In a size-based scheme for heterochrony (see page 42 in [26]) this would suggest that *Saimiri *evolved its small size by some sort of ontogenetic scaling or allometric progenesis or conversely, *Cebus *evolved its larger size by hypermorphosis, or both processes were involved since the genera diverged from their last common ancestor. At present is impossible to know the direction of change or in other words, which is the ancestral condition and which derived. Furthermore, both processes might have happen while both genera diverged from an ancestor of intermediate size.

Yet, despite conservation of allometric patterns, a broader, and perhaps more interesting, picture arises when we look at the comparison of growth trajectories (Table 5). Comparison of growth trajectories suggests two general and diverse underlying changes in development (Figure 6). Pattern one corresponds to an extension/truncation of the growth trajectory and occurs in 67% of all traits (Fig. 7a, b). Pattern two corresponds to a translation (see Fig. 4a in [31]) or height of otherwise parallel trajectories (Fig. 7c, d) and accounts for 23% of all traits. Therefore, developmental changes involved in the *Cebus*-*Saimiri *evolution seem to be to a larger part mix of two different heterochronic patterns: progenesis-hypermorphosis and pre-postdisplacement if we take a classic Morphometrics approach.

But, what light can be shed on this discussion by the results of the geometric morphometrics approach? First, is clear that the PC1-3D is an allometric size vector with a correlation of 0.97 with absolute size (centroid size). This is also the axis of major differentiation between the two genera and in fact the only one where they do not overlap and present a significant difference on a series of t-tests performed upon each of the first 40 PC's of the 3D analyses. These results are quite similar to the ones obtained with the canonical variate analyses done upon the original and the MASS data and basically reinforce the point that *Cebus *is a scaled-up version of *Saimiri*.

Also, the orientation of the PC1-3D is basically the same between the two genera (regression slope of PC1-3D against centroid size: k = 0.273, 95% CI 0.249–0.298 for *Cebus *and k = 0.265, 95% CI 0.217–0.314 for *Saimiri*). Second, PC2-3D is also an allometric vector with a high correlation with size if the focus is the within genus variation (r = 0.81 in *Cebus *and r = 0.70 in *Saimiri*). Indeed, PC2-3D is also highly correlated with PC1-3D (r = 0.77 in *Cebus *and r = 0.87 in *Saimiri*). Furthermore, the orientation of the PC2-3D is also basically the same between the two genera (regression slope of PC2-3D against size: k = 0.351, 95% CI 0.312–0.389 for *Cebus *and k = 0.293, 95% CI 0.231–0.356 for *Saimiri*). Considering all these results both PC's 3D can be interpreted as allometric vectors. In other words, PC2-3D represents ontogenetic allometry (Fig. 8) while PC1-3D represents evolutionary allometry (Fig. 7). In fact, changes in both PC's seem to some extent similar involving basically the neurocranium, face and zygomatic regions and it is not a surprise that both represent allometric variation.

What these results inform us about the evolution of *Cebus *and *Saimiri*? First, almost all differentiation between these two genera is related to size/scaling. Second, these differences either in scale (absolute size) or shape associated with size (allometry) follow a common evolutionary trajectory (Fig. 7). This last point is also totally supported by the correlation between absolute size, the axis of differentiation between the two genera (CV1), and PC1-3D (all R's larger than 0.98). Thirdly, ontogenetic variation in shape in one genus is parallel to the ontogenetic variation in the other. In other words, ontogenetic allometry follows a common and parallel trajectory between *Cebus *and *Saimiri *(Fig. 8) while the latter genus obviously start from a different point due to the huge difference in absolute size among them (*Saimiri *newborns weight 109 g while *Cebus *newborns weight 235 g). Fourthly, ontogenetic and evolutionary allometries are correlated. Considering all these findings together it seem correct to conclude that the evolution of these two genera results from developmental changes tweaking with body size and that nearly all differences observed among adult morphologies are a consequence of this size scaling. It is impossible at this time to polarize this change and in fact, it might well be the case that after the split from their common ancestor *Cebus *and *Saimiri *both diverges in opposite directions, *Cebus *scaling up and *Saimiri *scaling down.

What role might life-history evolution play in triggering those morphological changes? Figure 2 show that *Cebus *has a delayed on-set of reproduction. This is consistent with 67% of the traits growth trajectories and with the hypermorphic condition of *Cebus *and suggests that the extension of the growth trajectory was attained by delaying the age of first reproduction. Conversely, figure 3 shows that *Saimiri *neonates are born heavier than expected for a NWM of its size and this suggests that the height observed in the trajectories, where *Saimiri *is translated above *Cebus *(23% of all traits) might be explained by this larger starting point for the post-uterine growth period. Figure 4 also add another piece in this puzzle, showing that *Cebus *infants are weaned later than expected for a NWM of its size while the reverse is true for squirrel monkeys. Delayed weaning and age of first reproduction suggests that *Cebus *has a very slow developmental pattern compared to the rest of the NWM. Early weaning in *Saimiri *would suggest at first the reverse, but others factors should be considered here in judging whether or not *Saimiri *present a "fast" or " slow" life-history pattern. *Saimiri *neonates are born relatively heavy and represent almost 14% of the total weight of the mother, representing the largest pre-natal investment in NWM [11] in a single newborn (tamarins and marmosets which usually have twins invest even more if we consider litter weight). Squirrel monkey mothers also usually do not have support from group members in raising their infants which should impose a heavy burden on them. *Saimiri *compensate for this burden by a prolonged interbirth interval [11]. Garber and Leigh also point out that in *Saimiri *"An ontogenetic trajectory associated with large neonatal body size and rapid neurological development may facilitate early foraging independence, thus shifting metabolic costs away from the mothers and to the developing individual". After weaning, developing young follow a long and slow growth trajectory [11], which in a way is similar to their sister clade, *Cebus*. Figure 5 sheds additional light on this point, because the *Cebus*/*Saimiri *clade is characterized by the highest pre-natal growth rates among NWM, after accounting for differences in adult body size (and historical relatedness). Because most of neurocranial growth occurs during the pre- and peri-natal period, this faster growth in capuchins and squirrel monkeys accounts for the largest encephalization index in this clade within NWM [15], which can also be observed in the very long neural region in *Saimiri *and *Cebus *young's (Figures 7 and 8). Conversely, *Cebus *and *Saimiri *post-natal growth rates are among the lowest among NWM (Table 8). Therefore, summarizing all these life-history changes, the whole clade of capuchin/squirrel monkeys might be characterized by fast pre-natal growth and very slow post-natal growth. This is an interesting conclusion, because while obviously *Saimiri *represents a paedomorphic (juvenilized) morphology and *Cebus *a peramorphic (adult like) morphology when compared to one another, the whole clade might be considered paedomorphic relative to NWM as a whole.

**Table 8:** Life-history data

Genus	age first rep (days)	Adult Weight	Gestation Lenght	Age of weaning	Birth Weight	Pre-natal Growth Rate (regression)	Post-natal growth rate
Alouatta	1460.00	6404.2	186	369	407.7	1.150	1.337
Ateles	1642.50	8276.3	229	653	482.0	1.137	1.443
Brachyteles	2737.50	8840.0	225	639	.		
Lagothrix	2555.00	7150.0	218	340	450.0	1.135	1.124
Cacajao	1642.50	2893.8	180	547	.		
Chiropotes	1460.00	2632.5	160		.		
Pithecia	1125.42	2003.5	170	122	121.0	0.934	1.486
Callicebus	1350.50	997.3	160	192	100.0	0.907	1.078
Cebus	2007.50	2475.1	168	477	234.6	1.065	0.950
Saimiri	912.50	786.9	167	51	109.0	0.917	1.164
Aotus	730.00	1018.7	133	75	97.0	0.935	1.381
Leontopithec	638.75	471.4	133	91	50.0	0.800	1.430
Saguinus	699.58	444.4	145	79	43.0	0.756	1.482
Callimico	547.50	505.0	155	65	50.0	0.776	1.833
Callithrix	547.50	351.2	148	106	30.0	0.681	1.881
Cebuella	501.88	108.5	137	91	14.0	0.536	1.573

Age of first reproduction, Adult weight, gestation length, age of weaning, birth weight, and Pre- and Pos-natal growth rates are presented for NWM.

## Conclusion

*Saimiri *and *Cebus *represent a unique radiation within the NWM in many aspects. The differentiation of these two genera from their common ancestor is, to a large extent, due to size evolution. Most morphological differences between these two genera are related to scaling. Furthermore, this scaling is to a large extent due to a simple extension/truncation of growth, but also includes pre- and post-displacement. Several life-history changes seem correlated to, or perhaps are even causal of the morphological diversification of *Cebus *and *Saimiri*; such as delayed on-set of reproduction in *Cebus*, faster pre-natal growth rates and delayed weaning in *Cebus*, and accelerated weaning in *Saimiri*. Post-natal life-history is also slow in both genera relative to other NWM.

## Methods

### Sample and measurements

A total number of 886 specimens were measured, with 30 specimens not included in the analyses due to missing values. The adult sample includes 605 specimens in 18 species for the two genera as follows: 11 species of the genus *Cebus*, including the following species: *C. albifrons *(N = 13), *C. cesarae *(N = 17), *C. apella *(N = 135), *C. capucinus *(N = 20), *C. libidinosus *(N = 38), *C. macrocephalus *(N = 11), *C. nigritus *(N = 78), *C. nigrivitattus *(N = 9), *C. paraguayanus *(N = 19), *C. robustus *(N = 35), *C. xanthosternus *(N = 4); 7 species of the genus *Saimiri*, including the following species: *S. albigena *(N = 5), *S. boliviensis *(N = 6), *S. cassiquiarensis *(N = 29), *S. macrodon *(N = 13), *S. oerstedi *(N = 32), *S. sciureus *(N = 114), *S. ustus *(N = 16), and *S. vanzolinii *(N = 11). Adult specimens were used in the morphological differentiation analyses, properly controlling for species and sexual variation. Additional 41 specimens were discarded because they lost sex information (N_M _= 309 and N_F _= 255 for males and females).

An additional sample of 161 sub-adult and juveniles *Cebus *and 90 *Saimiri *skulls of varied age were also measured. Dental eruption sequence for all New World Monkeys was described in detail by [8]. I use the same developmental age (DA) criteria described in [30] and DA6 correspond to adult specimens. The following samples sizes were available for *Cebus*: DA1 (N = 25), DA2 (N = 22), DA3 (N = 41), DA4 (N = 13), DA5 (N = 60), DA6 (N = 379); and for *Saimiri*: DA1 (N = 9), DA2 (N = 2), DA3 (N = 4), DA4 (N = 10), DA5 (N = 65), DA6 (N = 226). Young samples (DA1 to DA4) not always present sex or species identification available and often lack any information regarding locality or accompanying skin that would allow proper identification of species and sex. The sampling here was as complete and throughout as possible but this lack of information result in a lack of power to perform growth analyses controlling for sexual and interespecific differentiation. However, most young and sub-adult samples (DA1 to DA5) are concentrated on two species, *Cebus apella *(99% of all specimens) and *Saimiri sciureus *(81%). Because the major goal here is to understand the differentiation and evolution of size and shape between the two genera the effect of uncontrolled sexual and specific variation within each genus would be to increase dispersion among points and consequently blur any observed pattern among genera. Results presented here are straightforward in this respect with a clear characterization of evolutionary and ontogenetic allometry (Figures 7 and 8) that seems robust for these other uncontrolled sources of variation (sex and species).

The specimens are deposited at the following institutions: American Museum of Natural History (AMNH), Museu de Zoologia da Universidade de São Paulo (MZUSP), Museu Nacional do Rio de Janeiro (MNRJ), Museu Paranaense Emílio Goeldi (MPEG) and National Museum of Natural History (USNM). A complete list of measured specimens sorted by taxon and museum collection may be obtained from the author upon request. Only adult crania were used in the subsequent analyses, except where specifically noted. Specimens were considered adult when they had fully erupted and functional dentition as well as closed or fused spheno-occipital and/or spheno-ethmoid sutures. Non-adult specimens correspond to a mixed age sample containing all tooth stages from a completely deciduous dentition to a permanent dentition except a functional canine or third molar [8].

**Figure 9:**
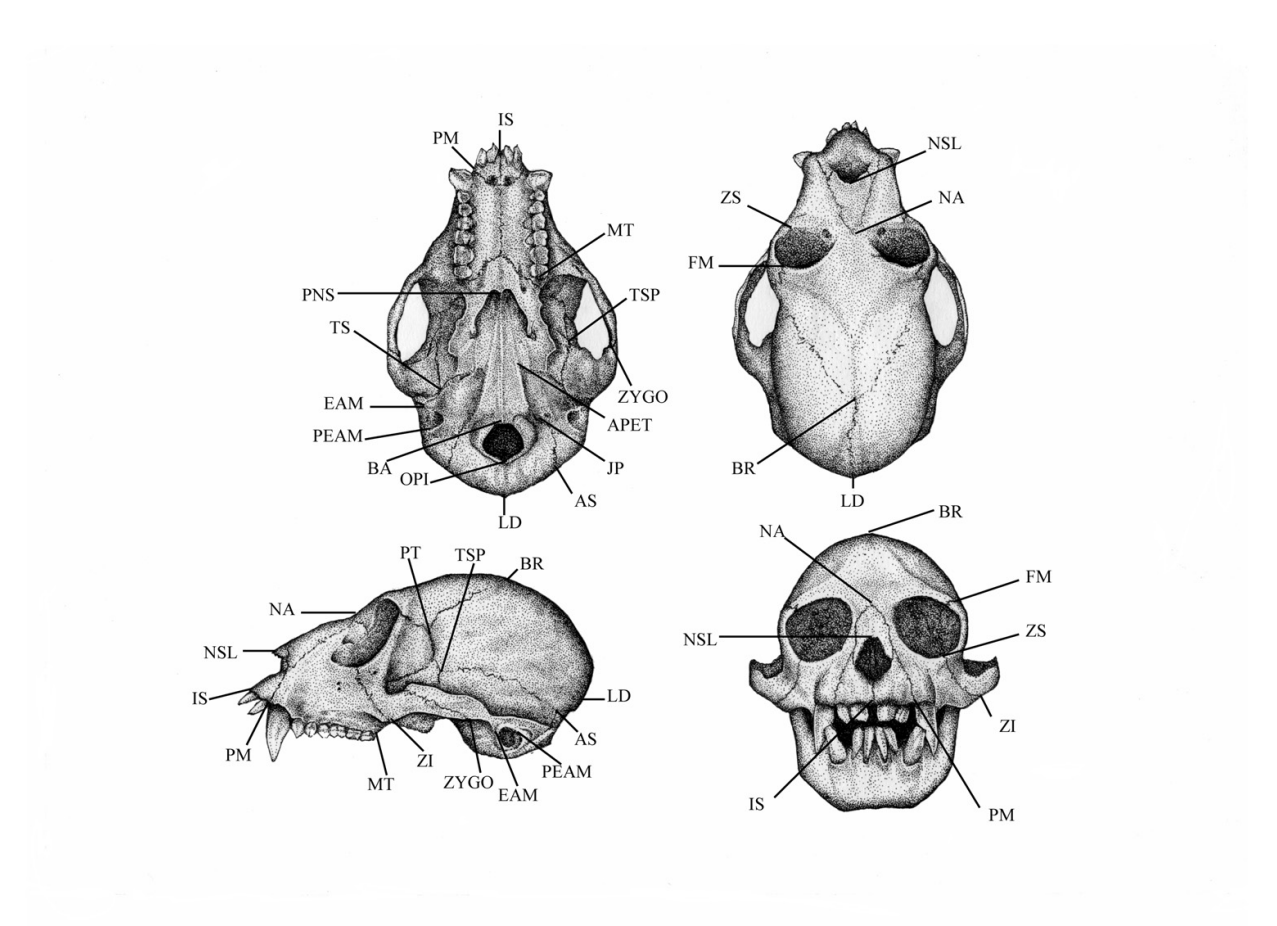
**New World Monkey skull with landmarks**. Craniofacial landmarks recorded from Cebinae skulls using three-dimensional digitizer. See Tables 9 and 10 for landmarks and measurements details.

Three-dimensional co-ordinates were recorded for 36 landmarks (Figure 9 and Table 9) using a Polhemus 3Draw or a Microscribe 3Dx digitizer. A small scale experiment was performed measuring a sub-sample of 20 specimens twice in each of the two digitizers. No significant differences were found between the digitizers. The general procedure for measuring specimens follows [6]. A set of 70 linear measurements describing cranial morphology was calculated from the co-ordinate values. This was reduced to a set of 39 measurements, after averaging measurements present on both sides of the skull (Tables 9 and 10). Whenever one of the skull sides was damaged, preventing me from taking any particular measurement, the other side is used. All results are presented in millimeters. All statistical analyses were performed using SYSTAT 11 (Richmond, CA).

**Table 9:** 22 Landmarks digitized

Landmark	Description	Position(s)	Order
IS	Intradentale superior, A	Midline	1
PM	Premaxillary suture at the alveolus, A	Right, left	2, 21
NSL	Nasale, A	Midline	3
NA	Nasion, A	Midline	4
BR	Bregma, AP	Midline	5
PT	Pterion, AP	Right, left	6, 22
FM	Fronto-malare, A	Right, left	7, 23
ZS	Zygomaxillare superior, A	Right, left	8,24
ZI	Zygomaxillare inferior, A	Right, left	9, 25
MT	Maxillary tuberosity, A	Right, left	10, 26
PNS	Posterior nasal spine, A	Midline	11
APET	Anterior petrous temporal, A	Right, left	12, 27
BA	Basion, AP	Midline	13
OPI	Opisthion, AP	Midline	14
EAM	Anterior external auditory meatus, A	Right, left	15, 28
PEAM	Posterior external auditory meatus, A	Right, left	16, 29
ZYGO	Inferior zygo-temporal suture, A	Right, left	17, 30
TSP	Temporo-spheno-parietal junction, A	Right, left	18, 31
TS	Temporo-sphenoidal junction at the petrous, AP	Right, left	19, 32
JP	Jugular process, AP	Right, left	20, 33
LD	Lambda, P	Midline	34, 35
AS	Asterion, P	Right, left	36

Landmarks recorded in Cebinae primates skulls using the three-dimensional digitizer. The designation A (anterior) or P (posterior) after the landmark name indicates in which position(s) the landmark was recorded. Landmarks are also identified in Figure 9. The order that each landmark was recorded is also presented (see additional movies material).

**Table 10:** 39 Linear distances and cranial regions

**Functional/Developmental group**	**Region**	**Trait**
Oral	Face	ISPM
Nasal	Face	ISNSL
Oral, nasal	Face	ISPNS
Oral	Face	PMZS
Oral	Face	PMZI
Oral	Face	PMMT
Nasal	Face	NSLNA
Nasal	Face	NSLZS
Oral, nasal	Face	NSLZI
Cranial vault	Neurocranium	NABR
Orbit	Neurocranium	NAFM
Nasal	Face	NAPNS
Cranial vault	Neurocranium	BRPT
Cranial vault	Neurocranium	BRAPET
Orbit	Neurocranium	PTFM
Cranial vault	Neurocranium	PTAPET
Cranial vault	Neurocranium	PTBA
Cranial vault	Neurocranium	PTEAM
Zygomatic	Face	PTZYGO
Cranial vault, zygomatic	Neurocranium, Face	PTTSP
Orbit	Neurocranium	FMZS
Zygomatic	Face	FMMT
Oral	Face	ZSZI
Oral	Face	ZIMT
Zygomatic	Face	ZIZYGO
Zygomatic	Face	ZITSP
Oral	Face	MTPNS
Cranial base	Neurocranium	PNSAPET
Cranial base	Neurocranium	APETBA
Cranial base	Neurocranium	APETTS
Cranial base	Neurocranium	BAEAM
Zygomatic	Face	EAMZYGO
Zygomatic	Face	ZYGOTSP
Cranial vault	Neurocranium	LDAS
Cranial vault	Neurocranium	BRLD
Cranial vault	Neurocranium	OPILD
Cranial vault	Neurocranium	PTAS
Cranial base	Neurocranium	JPAS
Cranial base	Neurocranium	BAOPI

Thirty-nine linear skull measurements (distances between landmarks) and membership in the six functional/developmental groups and two major cranial regions. Table 1 defines each landmark and Figure 9 shown their locations in a generalized Platyrrhine skull.

A total of 564 adult and 251 juveniles skulls with all 39 measurements (without missing values) were used in the analyses below. Juveniles were only used in the allometry analyses and were not included in the differentiation analyses. In this study I tested for differences between the taxa, the sexes and interaction between the sexes and taxa using multivariate analysis of variance (MANOVA). Given that squirrel and capuchin monkey species present sexual dimorphism with males usually larger than females, sexes were analyzed separately.

### Analyses

Interespecific Differentiation – Differences among Cebinae skulls were examined using the general linear model (GLM) module in SYSTAT 11 to perform a MANOVA and canonical variate analyses. Moreover, because the sampling includes several species within each genus and is not balanced in terms of the numbers of specimens per species, the MANOVA was performed for each sex with species nested within genus. In this way the between species variation within genus is accounted for so that the between genera differentiation is not inflated. Therefore the general linear model includes genus and species nested within genus as the two independent factors. For estimating the degree of differentiation among *Cebus *and *Saimiri*, Mahalanobis D^2 ^distances between group averages in the canonical function were calculated.

Allometry and scaling correction – The first principal component extracted from the ln-transformed data pooled within-group variance/covariance matrix of each genus and sex was computed. Because sexual variation in allometric patterns were small, detailed comparisons of allometric coefficients are presented only for the two genera. The thirty-nine standardized PC1 coefficient values of each group were divided by (1/√39) to assess divergence from isometry [16]. In order to compare allometric coefficients among Cebinae, it is important to determine the associated error of those values. A bootstrap procedure was used to set 95% confidence limits (L1 and L2) to the allometric coefficients (AC's) (see page 34 in [22]). A hundred bootstrap samples of N = 300 were taken and used to set up 95% confidence limits to AC's. Allometric coefficients with L2 below 1.0 were considered to be negatively allometric and conversely those AC's with L1 above 1.0 were considered positively allometric. For the juveniles a hundred bootstrap samples of N = 101 for *Cebus *and N = 26 for *Saimiri *were used to set 95% confidence limits to AC's.

The overall similarity of the allometric patterns is quantified with vector correlations, which measure similarity of vector orientation in a p-dimensional space (p being the number of traits). Vector correlations are equal to the cosine of the angle between vectors. The expected range of vector correlations commonly occurring among 39-element vectors by chance alone is -0.4 < r < 0.4 [1] with an average of 0.127 and a standard deviation of 0.095. Additionally, because there is a sampling error associated with each estimated allometric vector we use a self-correlation procedure to calculate allometric vector repeatability [6,23]. Allometric vector repeatability was estimated by correlating the observed PC1 and each of the 100 PC1 obtained from a bootstrap sample of replicates. These correlations provide a distribution of self-correlation [4]. The mean of this distribution is then used to measure allometry vector repeatability. To help judging how high allometric vector correlations are among genera and sex we adjust the observed vector correlations for estimation error by dividing the observed correlation by the square root of the product of the two vector repeatabilities (see [6,23]). I also use the strategy described by ([41], chapter 13, page 337) and compare each allometric vector to 100 random permutation of its elements. The rational underlying this approach is that if two vectors are "size" or "allometric" vectors with all elements positive, the range of vectors correlations is actually much smaller that from zero to one. Therefore every vector is permuted a 100 times and correlated with this random sample in order to test, using the corresponding average and confidence interval, whether or not correlation among any two vectors are indeed more similar that expected by chance alone.

I also used another strategy to analyze the relationship between size, shape and development based on [31] restriction of the term heterochrony and his focus on growth trajectories. Under this restriction heterochrony is a uniform change in the rate or timing of some ontogenetic process, with no change in the nature of the biological interactions going on within that process [31]. Uniform changes in the growth trajectory (trait × time) can be detected by comparing them (see Fig. 4 in [31]). One caveat in the analyzes performed here is that neither the *Saimiri *or *Cebus *data have time (age) available, given that the specimens were wild caught. Therefore, I plotted all 39 traits against skull size (all data ln-transformed in order to linearize the relationship), assuming that size is good proxy to time (see results for an indirect test of this assumption). To help visualize whether or not trajectories were linear and similar every plot included a LOWESS smooth function with tension equal to 0.3 (SYSTAT 11, Richmond, CA). The smoothing is produced by running along the X values and finding predicted values from a weighted average of nearby Y values. The surface is allowed to flex locally to better fit the data.

Given variation in squirrel and capuchin monkey size and, consequently, in allometric shape variation associated with those size differences, a normalization technique to scale data and remove allometric effects was applied [20,24]. This method, which I will refer from now on as "Multivariate Allometric Size-Scaling (MASS)", is derived from theoretical equations of allometric growth removing all the information related to size, not only scaling all individuals to the same size, but also adjusting their shape to account for allometry [20]. Here I follow Marroig and [24] modifying the [20] method by using the first principal component (PC1) score of the natural log data as the overall size measure and regressing all 39 traits onto PC1. The [20] correction is

Y*_i _= Y_i _[X_0_/X_i_]^b^

Where Y_i _and X_i _are the values of a specific trait and overall size (PC1 score) in individual 'i', respectively, Y*_i _is the theoretical value for the trait at the average size, X_0 _is the average antilog_e _of the PC1 scores, and 'b' is the PC1 coefficient for each of the 39 traits. Notice that 'b' is equal to the regression coefficient of the trait Y upon the PC1 scores. After this correction, the original data of all Cebinae are scaled to the same size, also adjusting their shapes for allometric scaling. These scale-corrected data were used to explore whether differences among *Saimiri *and *Cebus *were size dependent. This was done comparing the results of the MANOVA using the original (unscaled) and scale-corrected (MASS) data.

#### Geometric morphometrics

I also used a different approach to help visualize and test for differences in size and shape among *Cebus *and *Saimiri*. This geometric morphometrics approach was implemented using Morphologika, software developed by Paul O'Higgins and Nicholas Jones (University of York, see [27,7]). Detailed descriptions of Morphologika and the geometric Morphometrics theory can be found elsewhere [17,7,27,41]. The program uses generalized least squares superimposition to register landmark data. Registration is the basic procedure of translation, scaling, and rotation to remove all information unrelated to shape [41]. The resulting shape coordinates were subject to principal component analysis (PC's 3D from now on) in the tangent space (the Procrustes tangent projection) to Kendall's shape space [17,7]. What is important here is that this approach allows the separation of absolute size (scale differences quantified by the centroid size), shape differences due to allometry, and shape differences non-associated with size. A sample of 279 skulls was used in this analyses corresponding to all sub-adults and juveniles skulls and adults of the two most abundant species of each genus. Principal component scores were saved and used to test for differences as well as to interpret biologically each PC. One interesting feature in Morphologika is that the software allows the visualisation of the shape variability represented by the PCs which is achieved by reconstruction of the skulls (landmarks) in real time at any point along each PC axis.

The clear cut results in terms of separation and similarity between ontogenetic and evolutionary allometries (see below) arising from this geometric morphometric analysis, presents a new opportunity to develop a new approach to the study of allometry, growth and development. Landmarks configurations were obtained for each genus and age class along the PC1-3D and PC2-3D. The absolute differences between each of those average configurations represent the amount of changes occurring in each landmark along any period of the ontogeny. This allows a quantification of the magnitude of changes in each landmark throughout the ontogeny. Also, each of these differences between age classes defines a vector of changes in landmark position. Therefore is possible to quantify and compare those changes in shape using again vector correlation. These were calculated within each genus for consecutive age classes (age1-age2 × age2-age3, age2-age3 × age3-age4, and so on) as well as for similar age classes between genera (*Saimiri *age1-age2 × *Cebus *age1-age2, and so on). For those landmarks collected on both sides of the skull, the average of absolute magnitude of change was used in defining each vector. Therefore, each vector has 22 elements.

#### Life-history

I also obtained life-history data from the literature [15,11,9,19,28,37] for all New World Monkeys. Particularly, data on gestation length, body weight and skull size (my own observations from museum specimens, both skulls and labels), age at first reproduction, age at weaning, and birth weight, all transformed to natural log scale to make their relationships linear. Fetal growth rate was estimated by dividing the natural log of birth weight by the natural log of gestation length. Post-natal growth rate was estimated by regressing the natural log of adult weight by the natural log of the age of first reproduction (in days) and using the regression slope as an estimate of the rate (Table 8 show the life-history data). Association among these variables was tested using the independent contrasts (IC) method to account for the non-independence of phylogenetically structured data [12]. I use the module PDAP [12] within the MESQUITE package [21] to obtain the correlation among variables. The phylogenetic tree used is the same as in [25] based on [35]. Ideally, given that species within genus could vary in their life-histories, it would be necessary to correct for such differences properly accounting for phylogenetic relationships among species. Unfortunately robust and complete (with all species) phylogenetic hypotheses at the species within-genus level are not available for either *Cebus *or *Saimiri*. Also, not all species had life-history data available. These two pieces of information would be necessary to estimate ancestor values for the life-history traits. Therefore, in order to at least consider the range of variation in life-history among species within these two genera and check whether or not results from these analyses are consistent I use the minimum and maximum values for each life-history parameter to test the robustness of these regressions.

## Authors' contributions

Except for some young specimens measured by a colleague, GM is responsible for planning and executing all work involved in this paper.

## Supplementary Material

Additional file 1 3D animation of the morphometric analysis: Oblique view showing landmarks points (corresponding numbers in Table 9). x-axis represent the PC1-3D and the y-axis represent the PC2-3D. On the left are the points corresponding to Saimiri specimens and on the right those of Cebus. Symbols correspond to age classes, from age 1 (green diamonds) to age 6 (red cross). Click here for file

Additional file 2 3D animation of the morphometric analysis: Oblique view showing a wire frame connecting landmarks points.). x-axis represent the PC1-3D and the y-axis represent the PC2-3D. On the left are the points corresponding to Saimiri specimens and on the right those of Cebus. Symbols correspond to age classes, from age 1 (green diamonds) to age 6 (red cross). Click here for file

Additional file 3 3D animation of the morphometric analysis: Oblique view showing a surface reconstruction of the skull.). x-axis represent the PC1-3D and the y-axis represent the PC2-3D. On the left are the points corresponding to Saimiri specimens and on the right those of Cebus. Symbols correspond to age classes, from age 1 (green diamonds) to age 6 (red cross). Click here for file

Additional file 4 3D animation of the morphometric analysis: Lateral view showing landmarks points (corresponding numbers in Table 9). x-axis represent the PC1-3D and the y-axis represent the PC2-3D. On the left are the points corresponding to Saimiri specimens and on the right those of Cebus. Symbols correspond to age classes, from age 1 (green diamonds) to age 6 (red cross). Click here for file

Additional file 5 3D animation of the morphometric analysis: Lateral view showing a wire frame connecting landmarks points.). x-axis represent the PC1-3D and the y-axis represent the PC2-3D. On the left are the points corresponding to Saimiri specimens and on the right those of Cebus. Symbols correspond to age classes, from age 1 (green diamonds) to age 6 (red cross). Click here for file

Additional file 6 3D animation of the morphometric analysis: Lateral view showing a surface reconstruction of the skull.). x-axis represent the PC1-3D and the y-axis represent the PC2-3D. On the left are the points corresponding to Saimiri specimens and on the right those of Cebus. Symbols correspond to age classes, from age 1 (green diamonds) to age 6 (red cross).> Click here for file

Additional file 7 3D animation of the morphometric analysis: Dorsal view showing landmarks points (corresponding numbers in Table 9). x-axis represent the PC1-3D and the y-axis represent the PC2-3D. On the left are the points corresponding to Saimiri specimens and on the right those of Cebus. Symbols correspond to age classes, from age 1 (green diamonds) to age 6 (red cross). Click here for file

Additional file 8 3D animation of the morphometric analysis: Dorsal view showing a wire frame connecting landmarks points.). x-axis represent the PC1-3D and the y-axis represent the PC2-3D. On the left are the points corresponding to Saimiri specimens and on the right those of Cebus. Symbols correspond to age classes, from age 1 (green diamonds) to age 6 (red cross). Click here for file

Additional file 9 3D animation of the morphometric analysis: Dorsal view showing a surface reconstruction of the skull.). x-axis represent the PC1-3D and the y-axis represent the PC2-3D. On the left are the points corresponding to Saimiri specimens and on the right those of Cebus. Symbols correspond to age classes, from age 1 (green diamonds) to age 6 (red cross). Click here for file
